# Hot carrier extraction and ultrafast interfacial charge transfer in CsPbBr_3_/carbon dot nanocomposites

**DOI:** 10.1039/d6ra08246g

**Published:** 2026-10-08

**Authors:** Sukanya Saha, Guda Ramakrishna

**Affiliations:** a Department of Chemistry, Western Michigan University 1903 W Michigan Ave Kalamazoo MI 49008 USA sukanya.saha@wmich.edu

## Abstract

Interfacial thermalized and non-thermalized charge transfer between CsPbBr_3_ nanocomposites and carbon dots is studied using steady-state spectroscopy. Two types of carbon dots (CDs) were synthesized and characterized: one derived from amine-functionalized pyrene, and the other prepared using citric acid and *ortho*-phenylenediamine as precursor materials. Fourier transform infrared and X-ray photoelectron spectroscopy measurements have shown that the carbon dot synthesized from pyrene is amine-rich, whereas the carbon dot synthesized from citric acid is carboxylic acid-rich. The PL of CsPbBr_3_ nanocomposites is quenched by both CDs, indicating charge transfer between perovskite nanocomposites and the carbon dots. Transient absorption measurements have shown ultrafast charge transfer from perovskite nanocomposites to bound CDs from non-thermalized and thermalized excitonic states. We observed non-thermalized charge transfer from CsPbBr_3_ to CDs, with nearly 44% hot-hole transfer from CsPbBr_3_ to CD^−^_Pyr_ and 25% hot-electron transfer from CsPbBr_3_ to CD^−^_Cyan_. The charge transfer from thermalized states is monitored from the bleach recovery kinetics of CsPbBr_3_ with CDs, and the rates of charge transfer fall in the normal and inverted regions of the Marcus bell-shaped curve.

## Abstract 2

Schematic depicting perovskite-to-carbon-dot charge transfer *via* two competing pathways: thermalized (10^11^ s^−1^) and non-thermalized, hot-carrier transfer (10^12^ s^−1^), mediated by ligand exchange.

## Graphical abstract

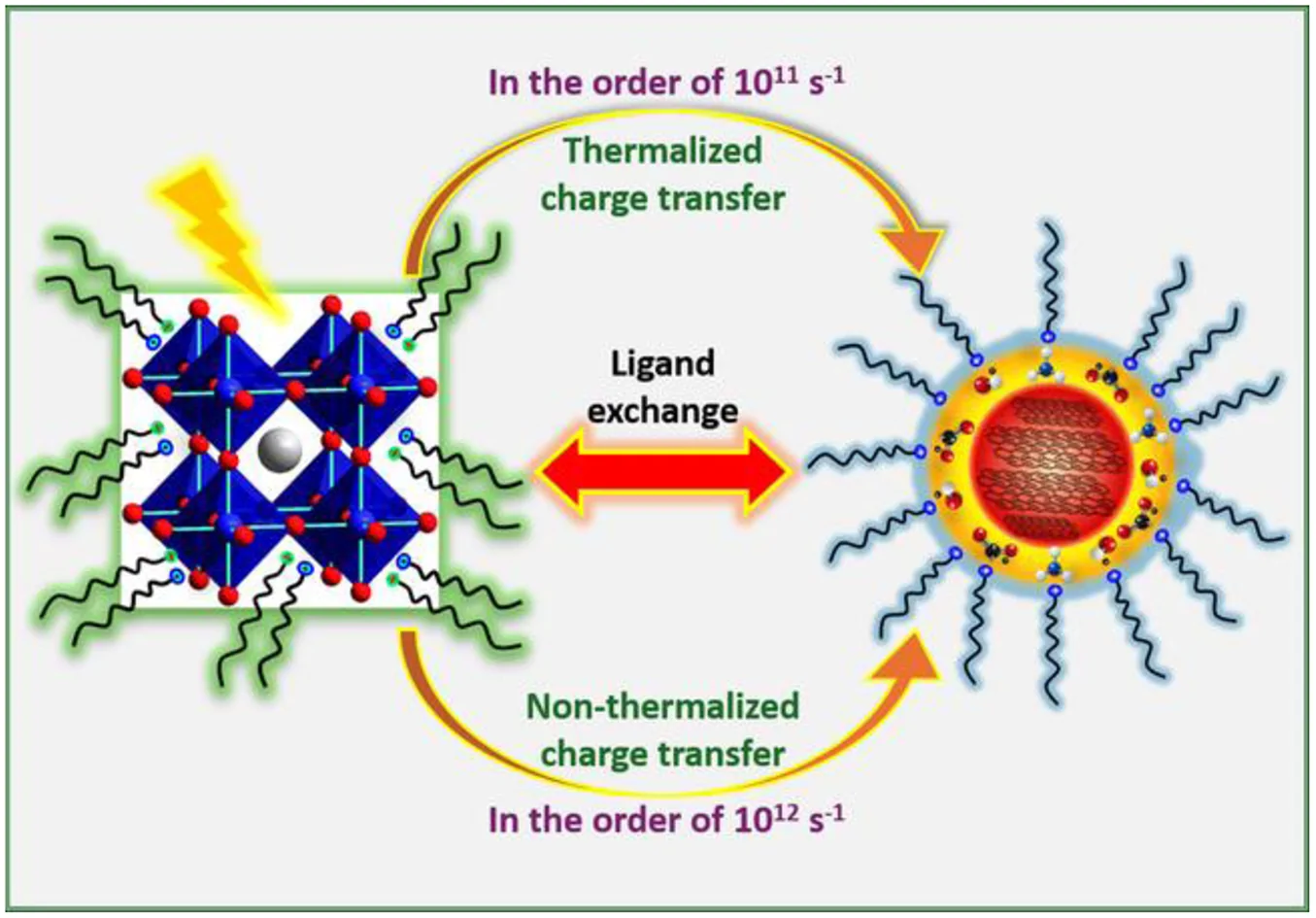

## 1 Introduction

Interfacial charge transfer between semiconductor nanomaterials and molecular adsorbates has been the topic of intense research interest for the last few decades.^1–5^ The understanding of charge transfer interactions has significant implications for their applications in optoelectronic devices^6^ such as photovoltaics, light-emitting diodes, optical sensors, *etc.* In this context, perovskite-based nanomaterials have gained significant research attention in recent years, both for their fundamental interest and optoelectronics applications.^1–7^ Research on semiconductor nanomaterials for optoelectronics applications has centred on two different strategies. Firstly, multi-exciton generation (MEG) in semiconductor nanomaterials,^8–11^ where quantum confinement increased the MEG efficiencies. Secondly, the extraction of energy from hot charge carriers in semiconductor nanomaterials.^12,13^ Excitation with photons of energies above the band edge creates electrons and holes that are non-thermalized.^14–16^ In most cases, these non-thermalized charge carriers relax to the band edge non-radiatively, thereby contributing to the loss of energy.^17^ Thus, the research has centred on exciting the hot charge carriers.^12,13^ Perovskite-based semiconductor nanomaterials show slowed-down hot carrier relaxation, thereby making them ideal candidates for harvesting non-thermalized charge carriers.^18–21^

Recently, significant work has focused on hot carrier (HC) cooling in lead halide perovskites, and pump-fluence-dependent transient absorption measurements are often used to monitor HC cooling dynamics.^18–23^ Electron and hole acceptors such as BQ and PTZ have long been used as selective probes of carrier transfer in quantum-confined semiconductors, including 0D CdSe quantum dots,^24^ 1D nanorods,^25^ and 2D nanoplatelets.^26^ These studies showed that ultrafast, carrier-selective transfer to molecular adsorbates depends on band alignment and on the degree of quantum confinement, which provides the framework later extended to perovskite nanocrystals. Sarkar *et al.* explored the interaction between CsPbBr_3_ NCs and BQ/PTZ. They observed a reduction in carrier density of CsPbBr_3_ NCs within 300 fs, indicating an ultrafast hot-carrier transfer process.^27^ Using Bphen (4,7-diphenyl-1,10-phenanthroline) as a hot-electron acceptor, Li *et al.* showed efficient hot-carrier extraction from MAPbBr_3_ nanocrystals.^28^ Using surface-anchored porphyrin molecules, Ghosh *et al.* found 45% hot-electron transfer efficiency from CsPbBr_3_ nanocrystals.^29^ The hot carrier extraction from MAPbI_3_ NCs has been shown by Kim *et al.* utilizing spiro-OMeTAD (a layer that transports holes) and TiO_2_.^30^ Pump-push-probe spectroscopy has been used to examine the hot carrier transfer from the more excited state of CH_3_NH_3_PbI_3_ to bathophenanthroline.^31^ Samanta and co-workers have demonstrated hot-hole transfer from CsPbBr_3_ to 4-mercaptophenol (MTH). The intrinsic slow cooling is attributed to the strong coupling between the NCs and the thiol functional group.^32^ Moreover, hetero-dimensional nanostructures have exhibited encouraging optoelectronic characteristics. Limited research has been documented regarding ultrafast investigations of CsPbX_3_ and heterostructure hybrid systems. Kamat *et al.* reported the use of a CsPbBr_3_/CdS heterostructure to enhance both stability and photocatalytic activity.^33^ Ghosh *et al.* used ultrafast transient absorption spectroscopy to investigate the dynamics of hot-electron transfer within the hybrid system of CsPbBr_3_ and thiol-functionalized reduced graphene oxide (rGo−Ph−SH) and reported 43% hot-electron extraction.^31^ Similarly, the group also reported CsPbBr_3_/PbSe heterostructure hot charge carrier extraction at low carrier density.^34^ In 2020, Rathore *et al.* documented the amalgamation of CsPbBr_3_ and N-doped carbon dots heterostructures in their publication. They observed a significant PL and photocurrent quenching in CsPbBr_3_/N-CDs.^35^ Burai *et al.* tuned charge-transfer efficiency through heteroatom doping (B, P) within a single CD platform.^36^ These studies have provided interesting insights into charge transfer dynamics across perovskite nanocrystal and carbon-dot interfaces.

In this study, charge transfer between perovskite nanocrystals and CDs has been investigated. The focus is to engineer the surface of CDs to be either electron-rich or electron-deficient and study how the nature of the CDs influences the interfacial charge transfer relaxation dynamics and hot carrier extraction.

## 2 Experimental

### 2.1 Materials

Lead bromide (PbBr_2_), cesium carbonate (Cs_2_CO_3_), 1-octadecene (ODE), oleylamine (OAm), and oleic acid (OA) were obtained from Sigma-Aldrich. All chemicals were of analytical reagent grade and used as received. Toluene served as the washing solvent during nanocrystal purification. The detailed synthetic procedure of CsPbBr_3_ nanocomposites and CDs is provided in the SI section.

### 2.2 Methods

Transmission electron microscopy (TEM) imaging was performed at the Centre for Nanoscale Materials using an accelerating voltage in the 100–300 keV range, with image acquisition carried out using a GATAN OneView digital camera. These measurements were used to resolve the particle morphology and lattice fringe spacings of the CsPbBr_3_ nanocomposites. X-ray Photoelectron Spectroscopy (XPS) measurements were conducted on a Kratos Axis Ultra spectrometer fitted with a monochromatic Al Kα X-ray source operated at 10 mA and 15 kV. All spectra were acquired under ultrahigh vacuum conditions maintained at a base pressure of 1 × 10^−9^ torr. Spectral deconvolution and binding energy calibration were performed using CasaXPS software.

Ground-state optical absorption and steady-state photoluminescence (PL) spectra were recorded on a Shimadzu UV-vis spectrophotometer and a Fluoromax-P photoluminescence spectrophotometer (Horiba Jobin Yvon), respectively. Surface functional group identification and vibrational mode assignments were carried out using a Shimadzu ATR-FTIR spectrometer. PL lifetime decay traces were measured by time-correlated single-photon counting (TCSPC) at a 370 nm excitation wavelength and a 1 MHz laser repetition rate.

Femtosecond transient absorption (TA) measurements were performed at Argonne National Laboratory on a Ti: Sapphire regenerative amplifier system (Spectra-Physics Spitfire Pro, 800 nm centre wavelength, 35 fs pulse duration, 6 mJ per pulse, 1 kHz repetition rate). Tunable pump pulses spanning 250 nm to 2.5 µm were generated using a TOPAS optical parametric amplifier (OPA), while the broadband probe continuum covering 420–800 nm was produced by white light continuum (WLC) generation. The pump and probe beams were spatially overlapped at the sample with their relative polarizations oriented at the magic angle, and the differential absorbance (Δ*A*) was recorded as a function of probe wavelength and pump–probe time delay. Raw TA datasets were subjected to group velocity dispersion chirp correction prior to kinetic fitting using Surface Xplorer software. The average number of photogenerated excitons per nanocrystal was controlled by systematic variation of the pump fluence, and continuous rotation of the sample holder was employed throughout data acquisition to prevent localized photodegradation. Variation of pump intensity allowed control over the number of excitons per nanocrystal, and sample holder rotation minimized optical damage during the experiment.

## 3 Results and discussion

### 3.1 Optical absorption and photoluminescence measurements

CsPbBr_3_ NCs were synthesized by the hot injection method, and the NCs were characterized with TEM; the edge length was determined to be 8 ± 0.4 nm (Fig. S1). The synthesized CDs were characterized by FTIR, Raman, and XPS measurements (Fig. S2–S5). From the analysis, CD^−^_Pyr_ was found to be amine-rich, while CD^−^_Cyan_ was found to be carboxylic-rich. Both show absorption below 400 nm and PL around 460 nm. To study the interaction of CsPbBr_3_ with CDs, it is necessary to solubilize them in the same solvent medium. CsPbBr_3_ is soluble in non-polar solvents such as toluene, while as-synthesized CDs are soluble in water. So, to study the interaction, CDs are phase-transferred from the aqueous phase to the organic phase by binding them with tetraoctylammonium bromide (TOA^+^Br^−^) as a surfactant.^37^

Optical absorption measurements are carried out to monitor the ground-state interaction between CsPbBr_3_ and CDs (Fig. 1A). The absorption spectrum of CsPbBr_3_/CD^−^_Pyr_ is the same as that of CsPbBr_3_. However, the absorption spectrum of CsPbBr_3_/CD^−^_Cyan_ is shifted to shorter wavelengths when compared to CsPbBr_3_. The shift is attributed to the binding of CD^−^_Cyan_ with CsPbBr_3,_ shifting the bandgap of the perovskite. Fig. 1B shows the PL spectra of CsPbBr_3_ with different concentrations (0–3.3 mg L^−1^) of CD^−^_Pyr_. Note from the figure that the PL of CsPbBr_3_ is quenched by adding CD^−^_Pyr_, indicating efficient excited-state interaction. Similarly, the PL of CsPbBr_3_ is also quenched with the addition of CD^−^_Cyan_. PL quenching typically arises from two alternative pathways: energy transfer or charge transfer. Non-radiative energy transfer is characterized by a decrease in donor emission accompanied by an increase in acceptor emission intensity. In our system, we observed PL quenching of CsPbBr_3_ upon addition of CDs, which is inconsistent with an energy transfer from perovskite to bound CDs, as there is limited spectral overlap between the CsPbBr_3_ emission and CD absorption (see SI). This behaviour therefore points to photoinduced charge transfer between perovskites and CDs.

**Fig. 1:**
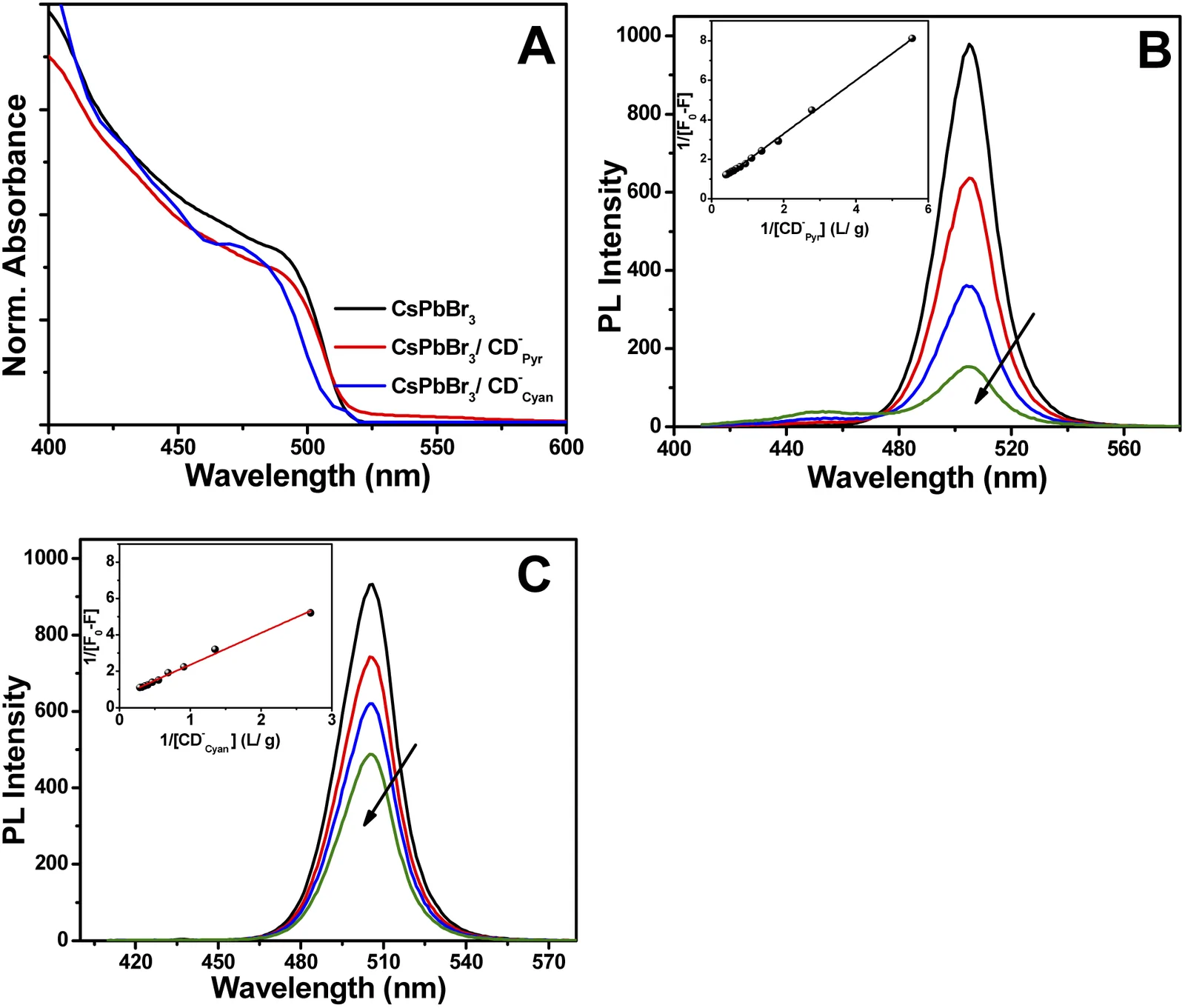
(A) Comparison of optical absorption spectra of CsPbBr_3_, CsPbBr_3_/CD^−^_Pyr_, and CsPbBr_3_/CD^−^_Cyan_. (B) PL of CsPbBr_3_ with increasing concentrations of CD^−^_Pyr_. The inset shows the Benesi–Hildebrand plot. (C) PL of CsPbBr_3_ with increasing concentrations of CD^−^_Cyan_. The inset shows the Benesi–Hildebrand plot.

To further understand the nature of quenching, time-resolved PL measurements were carried out using time-correlated single-photon counting, and the lifetime decreased with the addition of CD^−^_Pyr_ and CD^−^_Cyan_ (see Fig. S6 and Table S1). However, the lifetime quenching is smaller when compared to PL quenching, indicating that the quenching is static. Thus, the modified Benesi–Hildebrand equation was used to determine the association of CsPbBr_3_ with CDs, and the insets of Fig. 1B and C show the plots for CsPbBr_3_ with CD^−^_Pyr_ and CD^−^_Cyan_, respectively. The association constant of CsPbBr_3_/CD^−^_Pyr_ is determined to be 6.2 × 10^2^ L g^−1^, which is quite high compared to CsPbBr_3_/CD^−^_Cyan_ (3.5 × 10^1^ L g^−1^), indicating strong binding of CsPbBr_3_ with CDs. The maximum PL quenching is found to be 84% for the highest concentration of CD^−^_Pyr_ (at 3.3 mg L^−1^); on the other hand, the PL quenching is only 50% in CD^−^_Cyan_ at a similar concentration.

FTIR measurements are used to study the binding of CsPbBr_3_ and CD-hybrids, and a corresponding comparison of the IR spectra is shown in the SI. Lengthy oleate molecules envelop CsPbBr_3_'s surface, while CDs with negative charges are coated with the positively charged surfactant TOA^+^Br^−^. The surface of CsPbBr_3_ nanocrystals undergoes modification through a ligand exchange process in the presence of CDs, as confirmed by IR. Notably, the –OH stretching peak at 3500 cm^−1^ in CD^−^_Pyr_ shifted to 3330 cm^−1^ in CsPbBr_3_/CD^−^_Pyr_ (Fig. S7A). Similarly, the –COOH group-associated peak at 1770 cm^−1^ in CD^−^_Cyan_ shifted to 1725 cm^−1^ in CsPbBr_3_/CD^−^_Cyan_ (Fig. S7B). This study is consistent with the binding modes for CsPbBr_3_ with CD^−^_Pyr_, which are through the –OH bond, and CD^−^_Cyan_ attaches through the carboxylic acid group. As the CDs are bound to CsPbBr_3_, time-resolved measurements are carried out to monitor interfacial charge transfer between them.

Upon excitation of CsPbBr_3_ nanocrystals with photons with energies more than the band gap, electrons and holes in higher excitonic states are created. These hot carriers subsequently undergo relaxation to the band-edge through interactions involving carrier–carrier and carrier–phonon interactions, dissipating excess energy until reaching equilibrium temperature with the lattice. The overall charge carrier relaxation dynamics in CsPbBr_3_ NCs encompass exciton thermalization, biexciton recombination through Auger recombination, and exciton decay. One of the aspects of exciton dynamics in CsPbBr_3_ NCs is that they are pump-power dependent.^38,39^ Thus, power-dependent transient absorption is carried out to monitor interfacial charge transfer dynamics.

Femtosecond transient absorption measurements are used to study charge carrier relaxation dynamics of CsPbBr_3_ NCs in the presence of CD^−^ at different pump powers. The TA signal of the CDs alone is weak and featureless under comparable excitation (Fig. S8), and the features discussed below are dominated by CsPbBr_3_. At the lowest pump power (<*N*> ∼0.1, where <*N*> is the average number of absorbed photons per nanocrystal; see SI), CsPbBr_3_ has shown a growth of the bleach and long-lived decay (Fig. 2A). At this fluence, the probability of multi-exciton generation per nanocrystal is negligible, so band-edge state-filling scales directly with the number of photogenerated carriers, and Auger recombination effects (which dominate multi-exciton decay at higher fluences) do not contribute to the early-time dynamics. Parts B and C of Fig. 2 show the transient surface spectra of CsPbBr_3_ with CD^−^_Pyr_ and CD^−^_Cyan_ at <*N*> of 0.1, respectively. Note from Fig. 2B and C, the bleach recovered faster in the presence of CDs even at low pump fluence. The faster bleach recovery is attributed to interfacial charge transfer from CsPbBr_3_ to bound CDs. Transient absorption measurements on CsPbBr_3_ in the presence of CDs at high pump fluence have shown faster bleach recovery (see Fig. S9). The bleach recovery of CsPbBr_3_ at low pump power and high pump power is quite similar, suggesting that it is geminate recombination (see Fig. S10). The growth of the bleach signal in the spectra is used to determine the hot carrier cooling (HCC) time. Fig. 3A compares the growth rates of CsPbBr_3_ nanocrystals with the hybrid CsPbBr_3_/CD^−^. The growth time is shorter in CsPbBr_3_ than that of the pure system, showing hot-carrier transfer from pure CsPbBr_3_ nanocrystals to bound CD^−^. It is noteworthy that the carriers arrive at the band edge after the initial thermalization and relaxation phase, which causes both the expansion of the GSB and the decay of short-lived PIA decay to occur simultaneously. From the bleach recovery, the HCCs were determined and found to be (at <*N*> = 0.1) 550 ± 20, 320 ± 10, and 440 ± 10 fs for CsPbBr_3_, CsPbBr_3_/CD^−^_Pyr_, and CsPbBr_3_/CD^−^_Cyan_, respectively. Similar time constants were observed at high pump fluence (at <*N*> = 2.2). The obtained HCC of CsPbBr_3_ matched well with the literature.^39^ The non-thermalized charge-rate transfer is determined by the bleach growth time constant (Fig. 3A).1
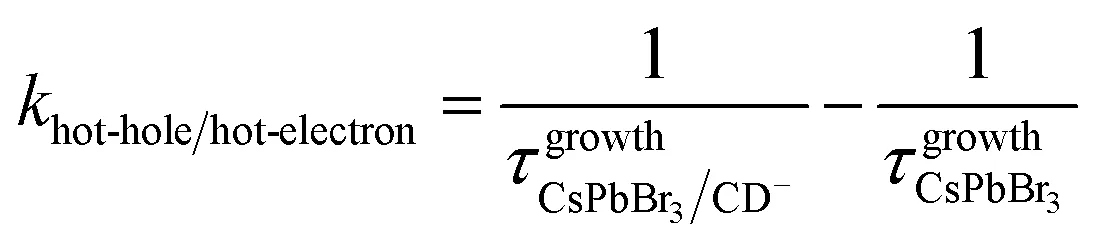


**Fig. 2:**
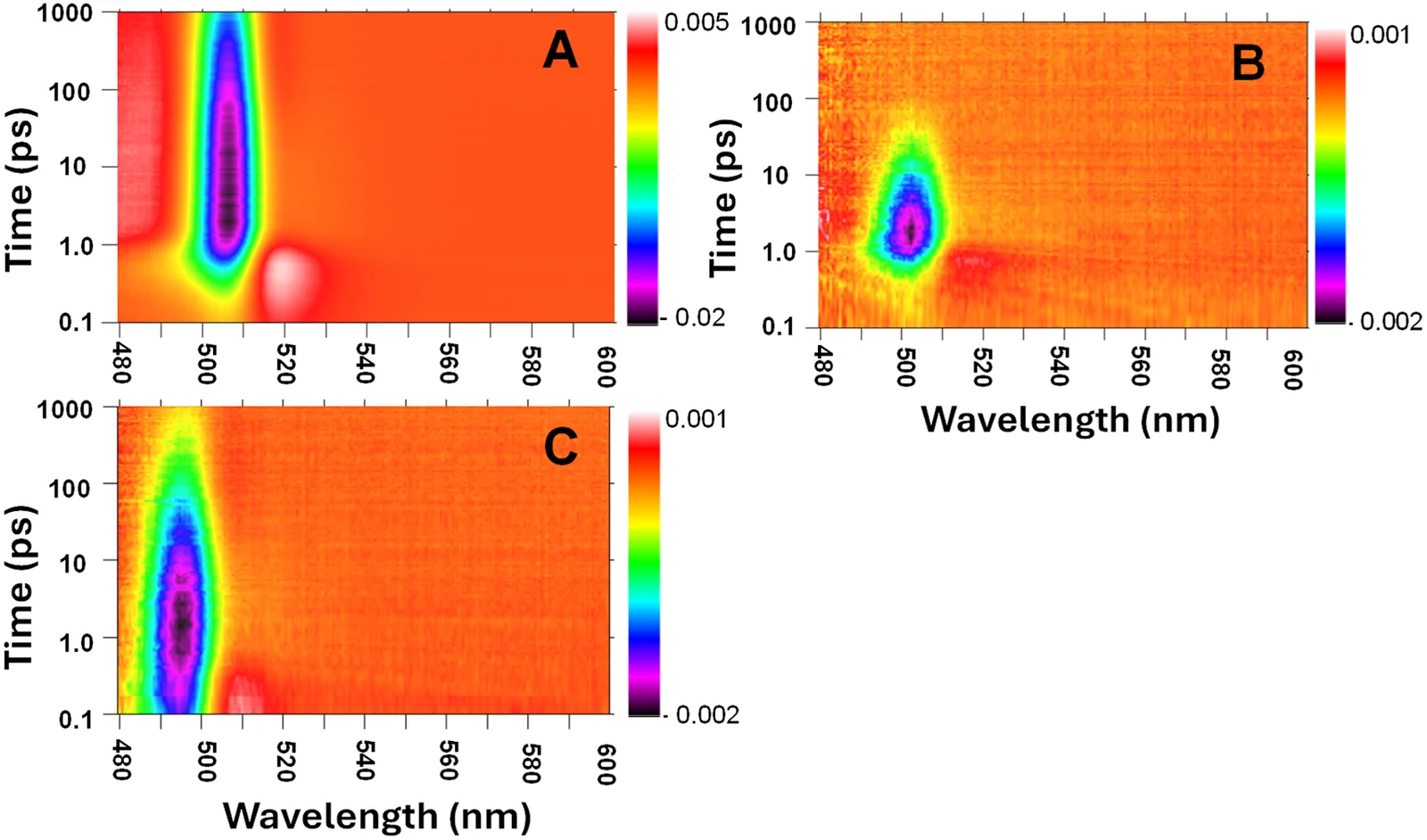
Transient absorption color mapping of (A) CsPbBr_3_, (B) CsPbBr_3_/CD^−^_Pyr_, and (C) CsPbBr_3_/CD^−^_Cyan_ at low <*N*> ∼0.1.

**Fig. 3:**
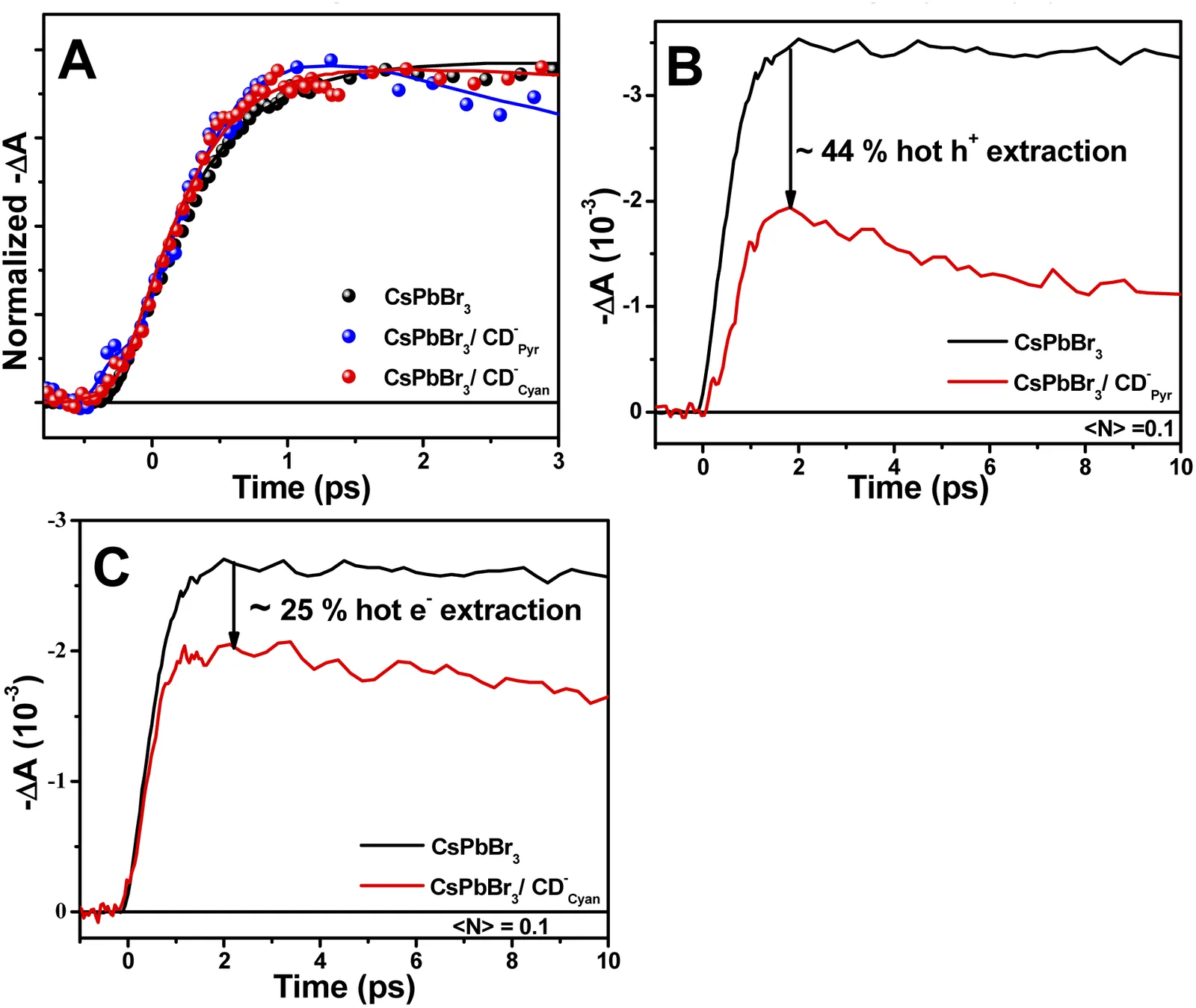
(A) Comparison of the growth of the bleach kinetics of CsPbBr_3_, CsPbBr_3_/CD^−^_Pyr_, and CsPbBr_3_/CD^−^_Cyan_. Absolute bleach amplitude comparison at early time scales between (B) CsPbBr_3_, CsPbBr_3_/CD^−^_Pyr_, and (C) CsPbBr_3_/CD_Cyan_^−^. *λ*_ex_ = 370 nm and <*N*> = 0.1.

The estimated hot-electron/hot-hole transfer rate from pure CsPbBr_3_ NCs to CD^−^ is found to be 1.31 × 10^12^ s^−1^ (for CD^−^_Pyr_) and 4.5 × 10^11^ s^−1^ (for CD^−^_Cyan_). The reason behind the charge transfer rate for carboxylated CD^−^_Cyan_ is lower because of the HOMO/LUMO alignment. The hot charge carrier transfer efficiency is also measured by monitoring the absolute bleach amplitude at early delay times.^32^ Interestingly, the significantly reduced amplitude suggests the transfer of hot-hole from pure CsPbBr_3_ NCs to CD^−^_Pyr_, and the hot-hole transfer efficiency is 44%, whereas the hot-electron transfer rate for CsPbBr_3_ NCs to CD^−^_Cyan_ is ∼25% (Fig. 3A–C). A similar trend is observed at higher pump power (see Fig. S11). The dynamics of the thermalized charge carrier transfer from pristine CsPbBr_3_ NCs to CD^−^ are analysed from the bleach recovery data. The kinetics of CsPbBr_3_ NCs is fitted with a decay component with a time constant >1 ns (100%), whereas the kinetics of CsPbBr_3_/CD^−^_Pyr_ and CsPbBr_3_/CD^−^_Cyan_ are fitted with the time constants 8 ± 0.1 ps (61.2%), 42 ± 2 ps (38.8%) and 27 ps (50%), 679 ps (50%), respectively. The calculated rate of band-edge electron transfer for CsPbBr_3_/CD^−^_Pyr_ and CsPbBr_3_/CD^−^_Cyan_ are 1.25 × 10^11^ s^−1^ and 3.7 × 10^10^ s^−1^. The rate of non-thermalized charge transfer is faster than thermalized charge transfer. Similarly, faster non-thermalized charge transfer is observed at high pump powers.

### 3.2 Interfacial charge transfer from thermalized states

Extraction of charge carriers from hot excitonic states was observed in CsPbBr_3_/CD^−^_Pyr_ and CsPbBr_3_/CD^−^_Cyan_. This phenomenon alone cannot explain the PL quenching of CsPbBr_3_ nanocomposites by both CsPbBr_3_/CD^−^_Pyr_ and CsPbBr_3_/CD^−^_Cyan_. Femtosecond transient absorption measurements have shown faster bleach recovery of CsPbBr_3_ nanocomposites by both CsPbBr_3_/CD^−^_Pyr_ and CsPbBr_3_/CD^−^_Cyan_, indicating charge transfer from the thermalized states of CsPbBr_3_ to bound CDs. Shown in parts A and B of Fig. 4 are the comparisons of bleach recovery kinetics of CsPbBr_3_, CsPbBr_3_/CD^−^_Pyr_, and CsPbBr_3_/CD^−^_Cyan_ at low pump fluence and high pump fluence, respectively. The bleach recovery of CsPbBr_3_ became faster in the presence of CDs and is attributed to interfacial charge transfer and charge recombination. The bleach recovery at low and high pump fluence is fitted with a multi-exponential decay, and the decay time constants obtained are provided in Table S2. The bleach recovery kinetics of CsPbBr_3_ alone are influenced by pump power, indicating that the charge carrier relaxation in perovskite nanocomposites involves the decay of biexcitons *via* Auger recombination at high pump powers, consistent with literature reports.^39^ In contrast, the bleach recovery of CsPbBr_3_ in the presence of CDs is not influenced by pump power, indicating that the charge carrier relaxation dynamics in the hybrid nanocomposites are dominated by interfacial charge transfer from CsPbBr_3_ to CD or *vice versa*. As the transient signals from the CDs are very low in the probe wavelength region, the CD radical-ion or any other intermediates were not observed.

**Fig. 4:**
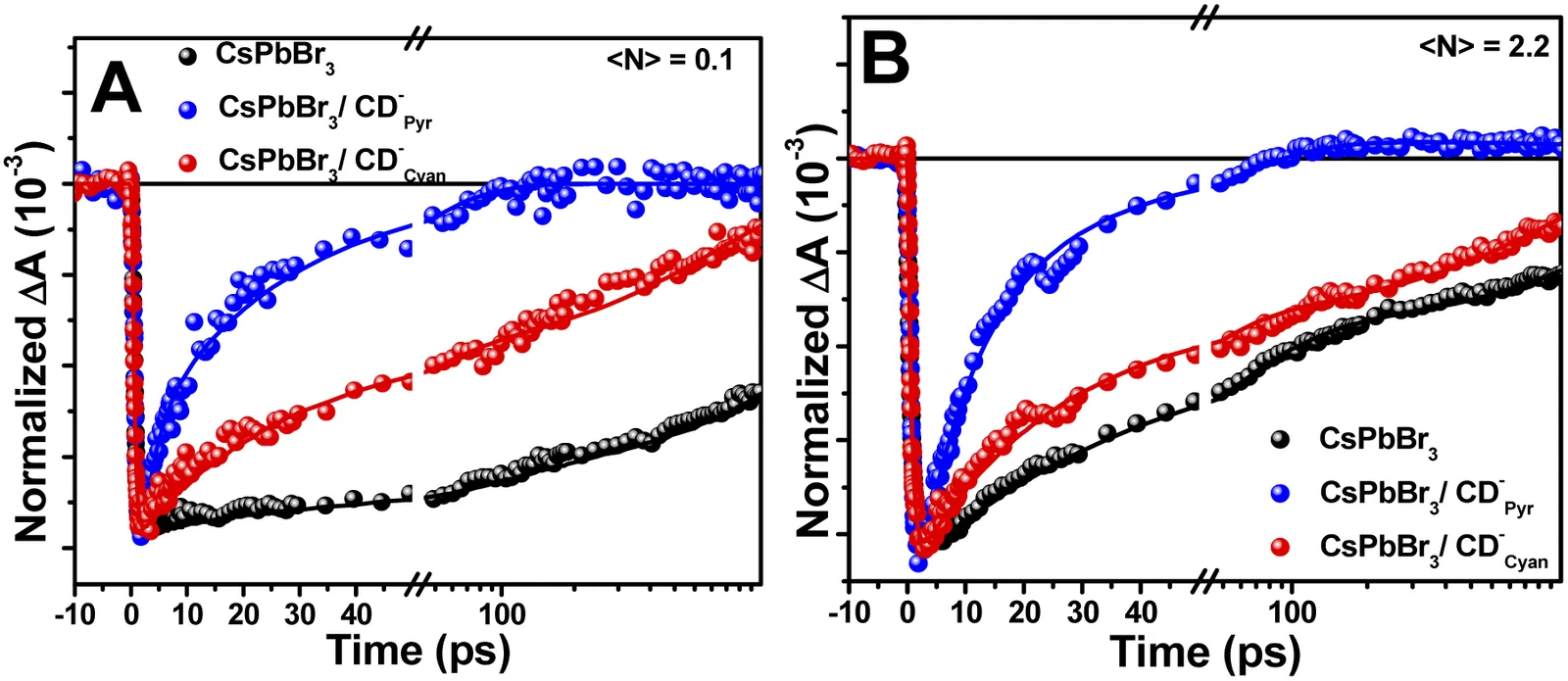
Comparison of bleach recovery kinetics of CsPbBr_3_, CsPbBr_3_/CD_Pyr_^−^, CsPbBr_3_/CD_Cyan_^−^ at (A) low pump fluence and (B) high pump fluence.

The CB edge and VB edge values of CsPbBr_3_ are obtained from the literature.^40^ The energy levels of CD^−^_Pyr_ and CD^−^_Cyan_ were determined from cyclic voltammetry (CV) measurements. We standardized the potentiostat using the Fe(CN)_6_^3−^/Fe(CN)_6_^4−^ system. CD^−^ was recorded in a three-electrode cell, with Pt as the counter electrode and Ag/AgCl/KCl as the reference electrode in an aqueous 0.1 M KCl electrolyte solution, on a CV-50 W equipped at a scan rate of 20 mV s^−1^. CV voltammogram was analysed and is provided in Fig. S13.

From electrochemical measurements, the band alignments of the CDs are determined and shown in Scheme 1. From the energy gaps, it can be seen that there is a hole transfer from CD^−^_Pyr_ to CsPbBr_3_ and electron transfer from CsPbBr_3_ to CD^−^_Cyan_.

**Scheme 1:**
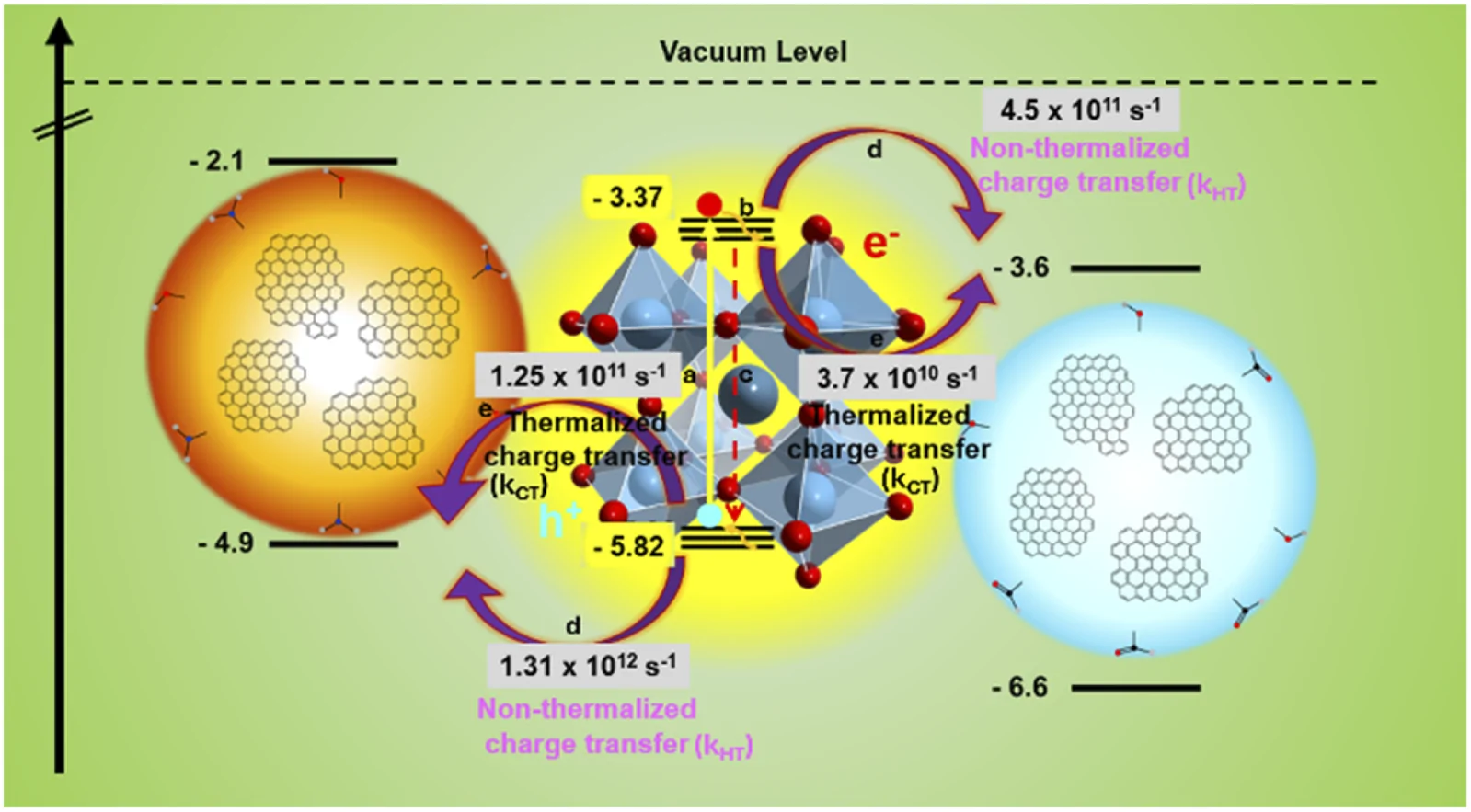
Schematic diagram depicting the energy levels and charge-transfer processes between CsPbBr_3_ and the carbon dots (CD^−^_Pyr_ on the left; CD^−^_Cyan_ on the right): (a) photoexcitation, (b) hot-carrier cooling (orange curved arrow), (c) charge recombination, (d) hot-carrier transfer (non-thermalized), and (e) (thermalized) charge transfer.

When the NCs are photoexcited, electrons from the valence band migrate to the conduction band, leaving holes in the valence band behind. The literature reports have shown that both the charge carriers, electrons and holes, in the CsPbBr_3_ NCs have nearly equal effective masses (
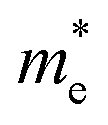
 = 0.15*m*_0_, and 
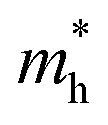
 = 0.14*m*_0_; *m*_0_ = rest mass of the electron), unlike cadmium QDs. It is impossible for there to be any electron transfer from the photoexcited CsPbBr_3_ NCs to any of the acceptor CDs since the band energy level alignment demonstrates that the VBM of the NCs resides below the HOMOs of each of the CDs. Nonetheless, hole transfer from the photoexcited CsPbBr_3_ NCs to CD^−^_Pyr_ and electron transfer to CD^−^_Cyan_ are thermodynamically feasible. For each CsPbBr_3_/CD^−^ hybrid, negative values of the Gibbs free energy change of charge transfer (Δ*G*) were obtained using the following formula:2
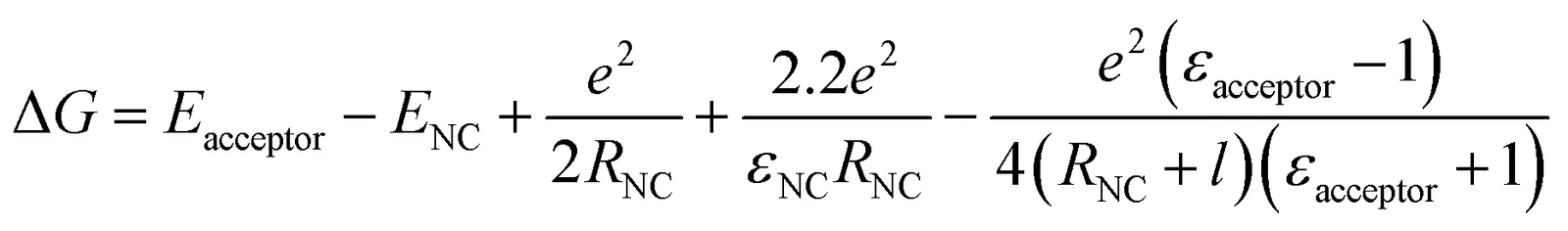


Utilizing eqn (2), the obtained Δ*G* value for CsPbBr_3_/CD^−^_Pyr_ is (−) 0.8 eV, while the value for CsPbBr_3_/CD^−^_Cyan_ is (−) 0.33 eV. Semiclassical Marcus theory states that the charge transfer rate (*k*_CT_) is represented by:3
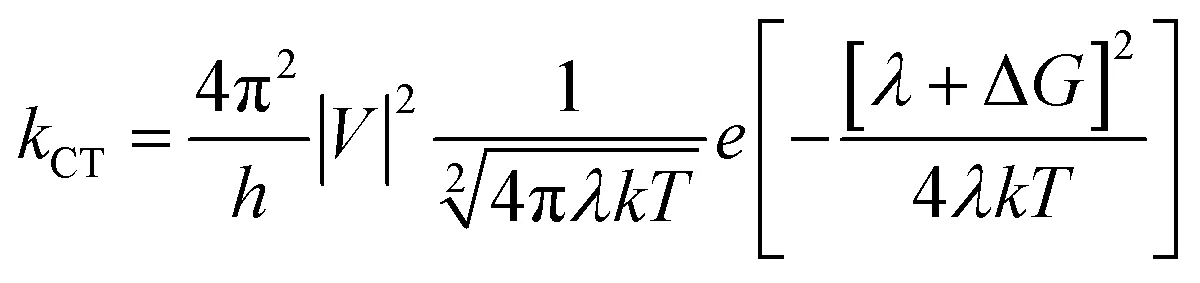
where *h* is the Planck constant, *V* denotes the electronic coupling between the initial and final states, −Δ*G* is the Gibbs free energy change (−Δ*G* is the driving force and normally positive), *k* is the Boltzmann constant, and *T* is the temperature. Typically, the overall reorganization energy for CT is between 0.4 and 0.6 eV; We fitted the forward and backward electron-transfer rates using eqn (3) and found *λ* = 0.83 eV. According to Fig. 5, the forward electron transfer for CsPbBr_3_/CD-hybrid falls under the normal region, and the backward electron transfer resides in the inverted region of Marcus theory.^40–42^

**Fig. 5:**
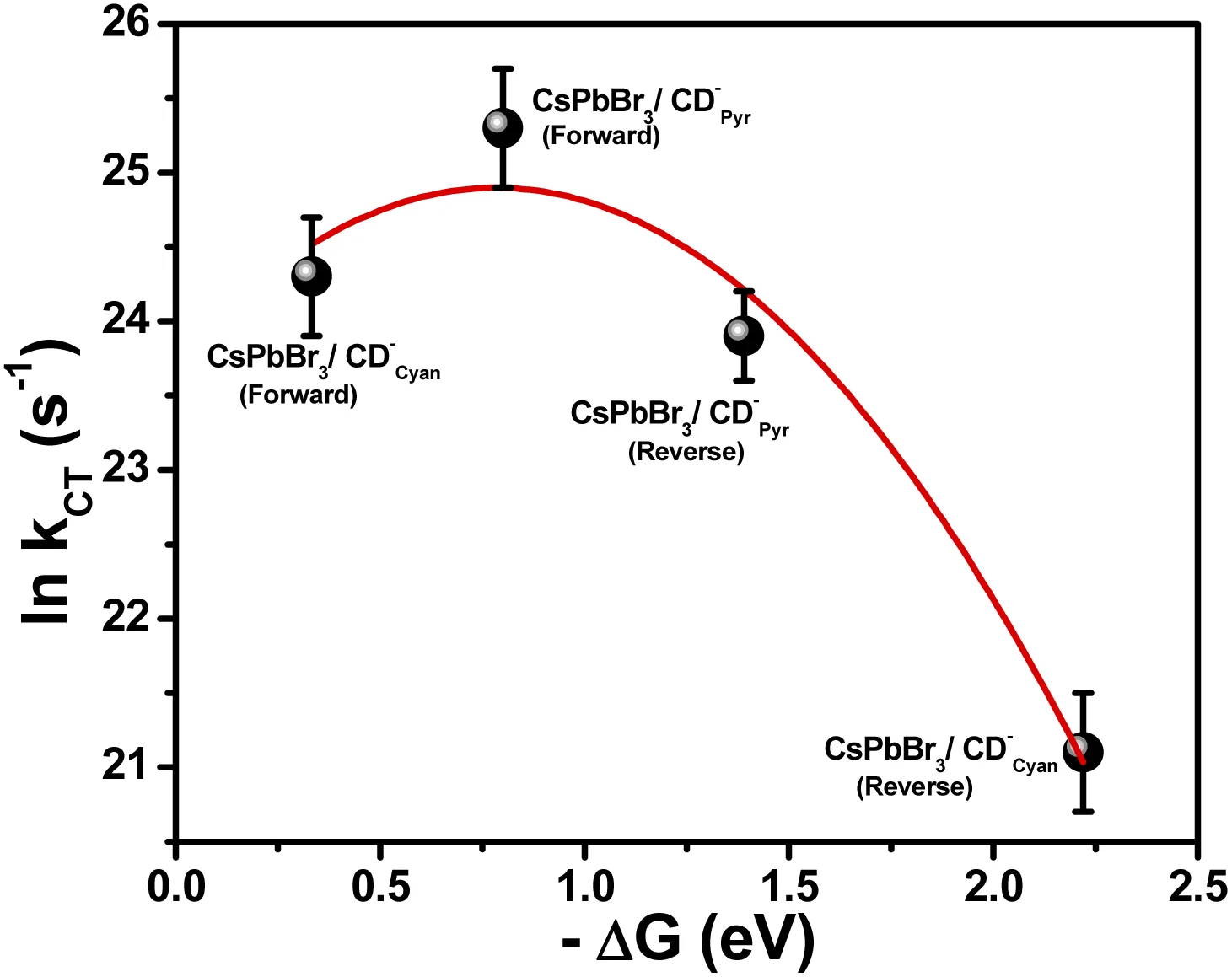
Plot depicting the rate of charge transfer *vs.* free energy. The red line is a guide to the eye.

## 4 Conclusions

Interfacial charge transfer from non-thermalized and thermalized states of CsPbBr_3_ to bound carbon dots was studied using steady-state and femtosecond transient absorption measurements. Carboxylic-acid and amine-functionalized carbon dots were synthesized and characterized using FTIR, Raman, and XPS measurements. The PL of CsPbBr_3_ nanocomposites is efficiently quenched by both CD^−^_Pyr_ and CD^−^_Cyan_. IR measurements have shown that CDs are bound to CsPbBr_3_. Femtosecond transient absorption measurements of CsPbBr_3_/CD heterostructures have shown faster bleach recovery of CsPbBr_3_ in the presence of CDs. Other alternative pathways could, in principle, also explain the faster bleach recovery, but none fit our data: energy transfer is unlikely given minimal CsPbBr_3_ emission-CD absorption overlap; surface trapping typically leaves an incomplete and long-lived recovery, whereas ours is complete. These observations ruled out the alternatives and point to interfacial charge transfer, governed by band alignment, as the mechanism most consistent with our data. Interestingly, charge extraction from non-thermalized excitonic states of perovskite nanocomposites to bound CDs was observed. Close to 44% hot charge carrier extraction was observed in CsPbBr_3_/CD^−^_Pyr_ while it is 25% for CsPbBr_3_/CD^−^_Cyan_. Ultrafast charge transfer from thermalized states of CsPbBr_3_ to bound CDs was observed, wherein the bleach recovery of CsPbBr_3_ perovskite nanocomposites became power-independent. The HOMO and LUMO energy levels of CDs were determined from combined cyclic voltammetry and steady-state PL measurements. The band alignment showed that electron transfer from the conduction band to CD^−^_Cyan_ was observed, while hole transfer from CsPbBr_3_ to CD^−^_Pyr_. From the bleach recovery kinetics, forward electron transfer and charge recombination time constants were determined. The obtained electron transfer rates follow the bell-shaped curve of theory, wherein the forward electron transfer is in the normal region while charge recombination is in the inverted region.

## Author contributions

S. Saha conceptualized and designed the study, performed the investigation and formal analysis, curated the data, prepared the visualizations, and wrote the original draft of the manuscript. R. Guda supervised the project, acquired funding, contributed to the conceptualization, and reviewed and edited the manuscript.

## Conflicts of interest

There are no conflicts to declare.

## Supplementary Material

RA-OLF-D6RA08246G-s001

## Data Availability

Supplementary information (SI): the synthesis procedures, additional optical (UV-vis, PL) and structural characterization (TEM, Raman, FTIR, XPS), time-resolved PL and transient absorption data, cyclic voltammetry, and the calculation of the average number of absorbed photons per nanocrystal. See DOI: https://doi.org/10.1039/d6ra08246g.
