# Supplementary material for: Hot carrier extraction and ultrafast interfacial charge transfer in CsPbBr3/carbon dot nanocomposites
Source: RSC Adv. 2026 Oct 8;16(46):60848–54. doi: 10.1039/d6ra08246g (PMC13646728; doi:10.1039/d6ra08246g)
Supplement: Supplementary file 1 [file RA-016-D6RA08246G-s001.pdf]

Supplementary Information (SI) for RSC Advances.
This journal is © The Royal Society of Chemistry

Supplementary information for

Hot Carrier Extraction and Ultrafast Interfacial Charge Transfer in $CsPbBr_3$/Carbon Dots Nanocomposites

Table of Contents

1. Synthesis of $CsPbBr_3$ Nanocomposites
2. Synthesis of Aminated CDs (CD^{-}_{Pyr})
3. Synthesis of N-doped Carboxylated CDs (CD^{-}_{Cyan})
4. Optical Absorption and PL of the Synthesized $CsPbBr_3$ Nanocrystals

   Figure S1. Optical absorption/ steady-state PL of $CsPbBr_3$ – The Inset of the Figure shows the TEM and histogram of the size
5. Optical and Structural Properties of Synthesized Carbon Dots

   Figure S2. (A) Optical absorption, (B) Raman, and (C) FTIR spectra of CD^{-}_{Pyr}

   Figure S3. (A) Optical absorption, (B) Raman, and (C) FTIR spectra of CD^{-}_{Cyan}
6. X-ray Photoelectron Spectroscopy (XPS) of Synthesized Carbon Dots

   Figure S4. XPS of CD^{-}_{Pyr}

   Figure S5. XPS of CD^{-}_{Cyan}
7. Time-Resolved PL Kinetics Decay Traces

Figure S6. PL kinetic decay traces of $CsPbBr_3$ with different concentrations of (A) CD^{-}_{Pyr}, and (B) CD^{-}_{Cyan} (λ_{ex}= 370 nm and λ_{em}= 506 nm).

Table S1. Time-resolved PL decay parameters of pure $CsPbBr_3$ nanocrystals and (A) $CsPbBr_3$/ CD^{-}_{pyr} and (B) $CsPbBr_3$/ CD^{-}_{Cyan} (λ_{ex}=370 nm and λ_{em}= 506 nm).

8. FTIR Measurements

Figure S7. Comparison of FTIR spectra of $CsPbBr_3$, CD, and $CsPbBr_3$/CD hybrids for (A) CD^{-}_{Pyr}, and (B) CD^{-}_{Cyan}.

9. Transient Absorption Measurement

Figure S8. Excited-state absorption spectra at different time delays of (A) CD^{-}_{Pyr}, (B) CD^{-}_{Cyan} in toluene at 370 laser excitation, <N> = 3.6.

Figure S9. Transient absorption color mapping of (A) $CsPbBr_3$, (B) $CsPbBr_3$/CD^{-}_{Pyr}, and (C) $CsPbBr_3$/CD^{-}_{Cyan} at high pump fluence (<N> = 2.2)

10. Bleach Recovery Kinetics

Figure S10. Comparative Analysis of Bleach Recovery Kinetics in $CsPbBr_3$, $CsPbBr_3$/CD^{-}_{Pyr}, and $CsPbBr_3$/CD^{-}_{Cyan} under different pump fluences.

11. Bleach Formation Kinetics

Figure S11. (A) Comparison of the bleach formation kinetics, and (B, C) Absolute bleach amplitude comparison at early time scales between $CsPbBr_3$, $CsPbBr_3$/$CD^{-}_{Pyr,}$ and $CsPbBr_3$/CD^{-}_{Cyan}. λ_{ex}= 370 nm and <N> = 2.2.

Table S2. Bleach formation time for $CsPbBr_3$ NCs, $CsPbBr_3$/ CD^{-}_{Pyr} and $CsPbBr_3$/ CD^{-}_{Cyan} NCs at 370 nm laser excitation and different pump powers.

12. Species-Associated Spectra

Figure S12. Species-associated spectra obtained from global fit analysis at low and high pump power for (A, D) $CsPbBr_3$, (B, E) $CsPbBr_3/CD^-_{Pyr}$, and (C, F) $CsPbBr_3/CD^-_{Cyan}$.

13. Cyclic Voltammetry

Figure S13. Cyclic voltammetry of (A) CD^-_{Pyr}, and (B) CD^-_{Cyan}

14. Spectral Overlap of Donor Emission and Acceptor Absorption

Figure S14. Spectral overlap between $CsPbBr_3$ emission and CD absorption

15. Calculation of average number of absorbed photons per nanocrystal

1. Synthesis of Perovskite Nanocomposites

(A) Preparation of Cesium Oleate

Cs_2CO_3 (2.5 mmol) was combined with ODE (40 mL) and OA (2.5 mL) in a two-neck 100 mL round-bottom flask. The mixture was degassed under vacuum with continuous stirring at 120 °C for 60 mins. The temperature was subsequently raised to 150 °C and maintained until complete dissolution of the carbonate salt, confirming quantitative formation of cesium oleate. The resulting solution was kept warm (≥100 °C) prior to injection to prevent precipitation.

(B) Preparation of Cesium Lead Bromide Nanocrystals

$CsPbBr_3$ nanocrystals were prepared by a solution-phase hot-injection route. $PbBr_2$ (0.376 mmol) was dispersed in ODE (10 mL) in a two- or three-neck flask and degassed under dynamic vacuum at 120 °C for 1 h. Dried OAm (1 mL) and OA (1 mL) were then introduced into the flask under a nitrogen atmosphere. Once the lead salt had fully dissolved, the reaction temperature was increased to 180 °C. Pre-warmed cesium oleate solution (4.4 mL, equilibrated at 100 °C) was

rapidly injected, and the flask was immediately immersed in an ice-water bath to terminate nanocrystal growth.

(C) Isolation and Purification of $CsPbX_3$ Nanocrystals

Immediately upon adding Cs-oleate, a precipitate is formed in the solution. The solution is transferred to 50 mL centrifuge tubes. The solutions were centrifuged at 25 °C and 10000 rpm for 10 min using a Sorvall Lynx 4000 centrifuge. The supernatant was discarded, and the nanocrystals were redispersed in hexane. To redisperse the nanocrystals, the precipitate was sonicated for 1 h using a Branson 1510 sonicator. Following sonication, the solution was centrifuged for 5 mins at 23 °C and 3000 rpm. The resulting supernatant was the purified colloidal solution of perovskite nanocrystals. [1]

2. Synthesis of Aminated CDs (CD^-_{Pyr})

CD^-_{Pyr} was synthesized via a hydrothermal carbonization route using nitropyrene as the molecular precursor.[2] Briefly, finely ground pyrene powder was combined with 20 mL of concentrated nitric acid (70% HNO_3) in a round-bottom flask to yield 1,3,6-trinitropyrene through nitration. The resulting solid product was isolated by vacuum filtration and dried overnight under ambient conditions. The dried 1,3,6-trinitropyrene was subsequently transferred to a sealed stainless-steel autoclave and subjected to hydrothermal treatment at 200 °C for 14 hours. The autoclave was then allowed to cool naturally to room temperature. The resulting yellow, fluorescent CDs solution was separated from unreacted carbonaceous residues by vacuum filtration through a 0.1 µm membrane filter and subsequently stored for further characterization and use.

3. Synthesis of N-doped Carboxylated CDs (CD^-_{Cyan})

N-doped carboxylated carbon dots (CD^{-}_{Cyan}) were prepared following a previously reported hydrothermal procedure.[3] In a typical synthesis, o-phenylenediamine (oPD, 108 mg) and citric acid (CA, 192 mg) were dissolved in 10 mL of redistilled water in a beaker. The mixture was sonicated until complete dissolution of both precursors was confirmed visually. The solution pH was adjusted to and maintained at 1 before transferring into a 100 mL stainless-steel autoclave. The sealed autoclave was heated at 140 °C for 6 hours, after which it was allowed to cool to room temperature naturally before the product was recovered.

To obtain negatively charged CDs (CDs^{-}; pH ≈ 10 as obtained after synthesis), the sample was transferred to a beaker. Throughout the procedure, the pH was continuously monitored using a pH meter, while the solution was vigorously stirred to maintain uniform pH conditions throughout the system.

Purification Process of CDs

The obtained CDs were dialyzed for 24 hours using a 1 kDa membrane. Water-soluble CDs were transferred to the organic phase (toluene) using a positively charged surfactant, tetra-octyl ammonium bromide (TOABr).

Synthesis of $CsPbBr_3$/ CDs Nanocomposites

$CsPbBr_3$/CD hybrid was synthesized using a straightforward ligand-exchange method. We created several solutions by adding increasing concentrations of CD^{-}_{Pyr} and CD^{-}_{Cyan} to a constant concentration of pristine $CsPbBr_3$ NCs. The final solution mixture was stirred for 10 minutes.

4. Optical Absorption and PL of the Synthesized $CsPbBr_3$ Nanocrystals

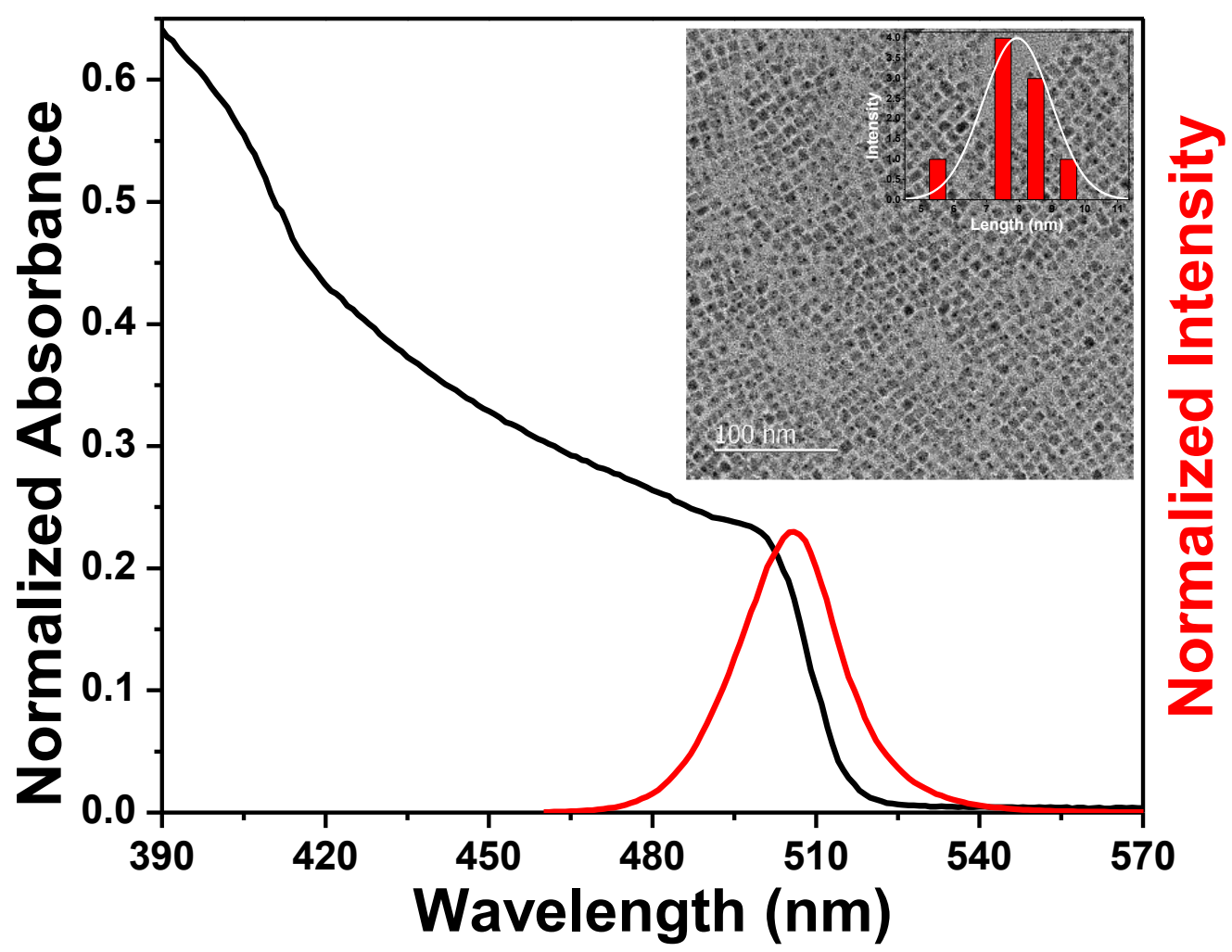

Figure S1. Optical absorption/ steady-state PL of $CsPbBr_3$ – The Inset of the Figure shows the TEM and histogram of the size.

5. Optical and Structural Properties of Synthesized Carbon Dots

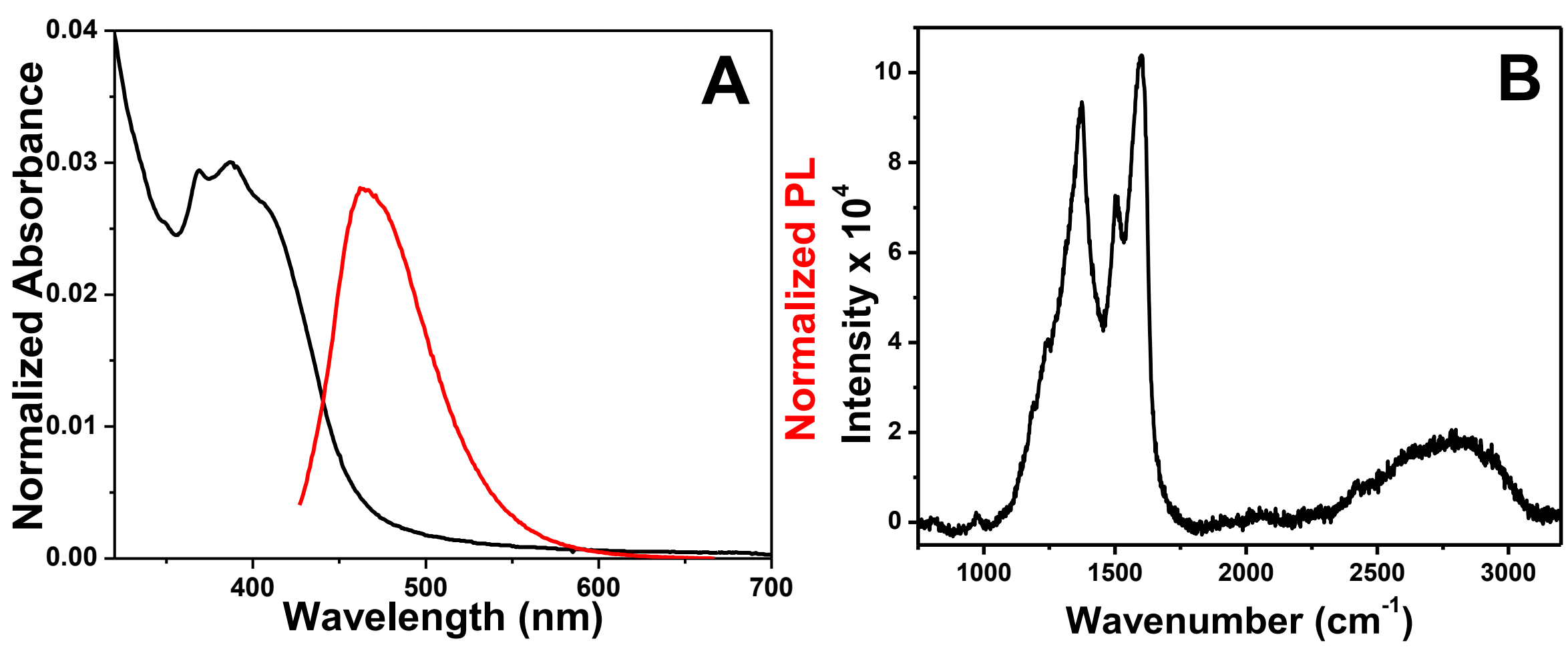

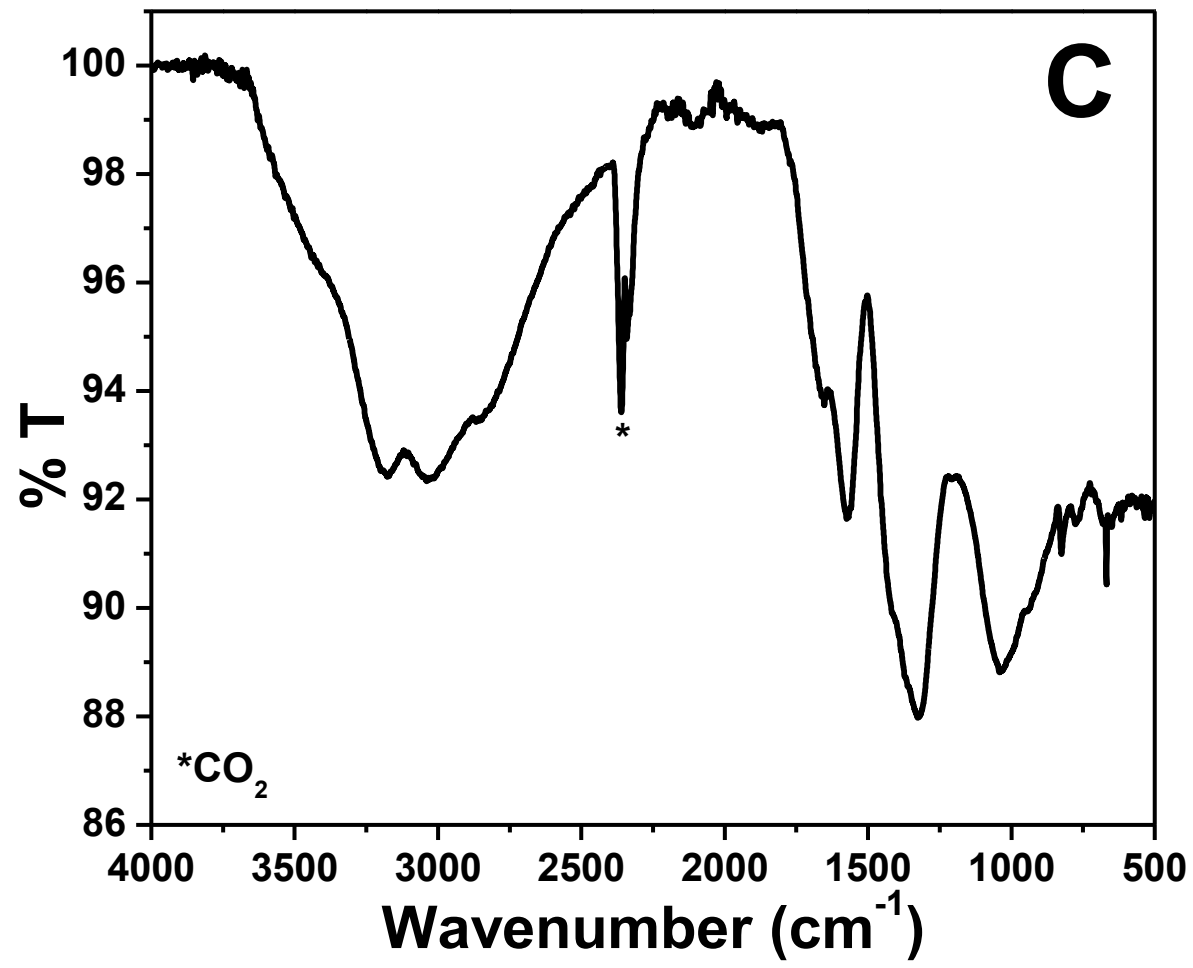

Figure S2. (A) Optical absorption, (B) Raman spectra, and (C) FTIR of CD^{-}_{Pyr}

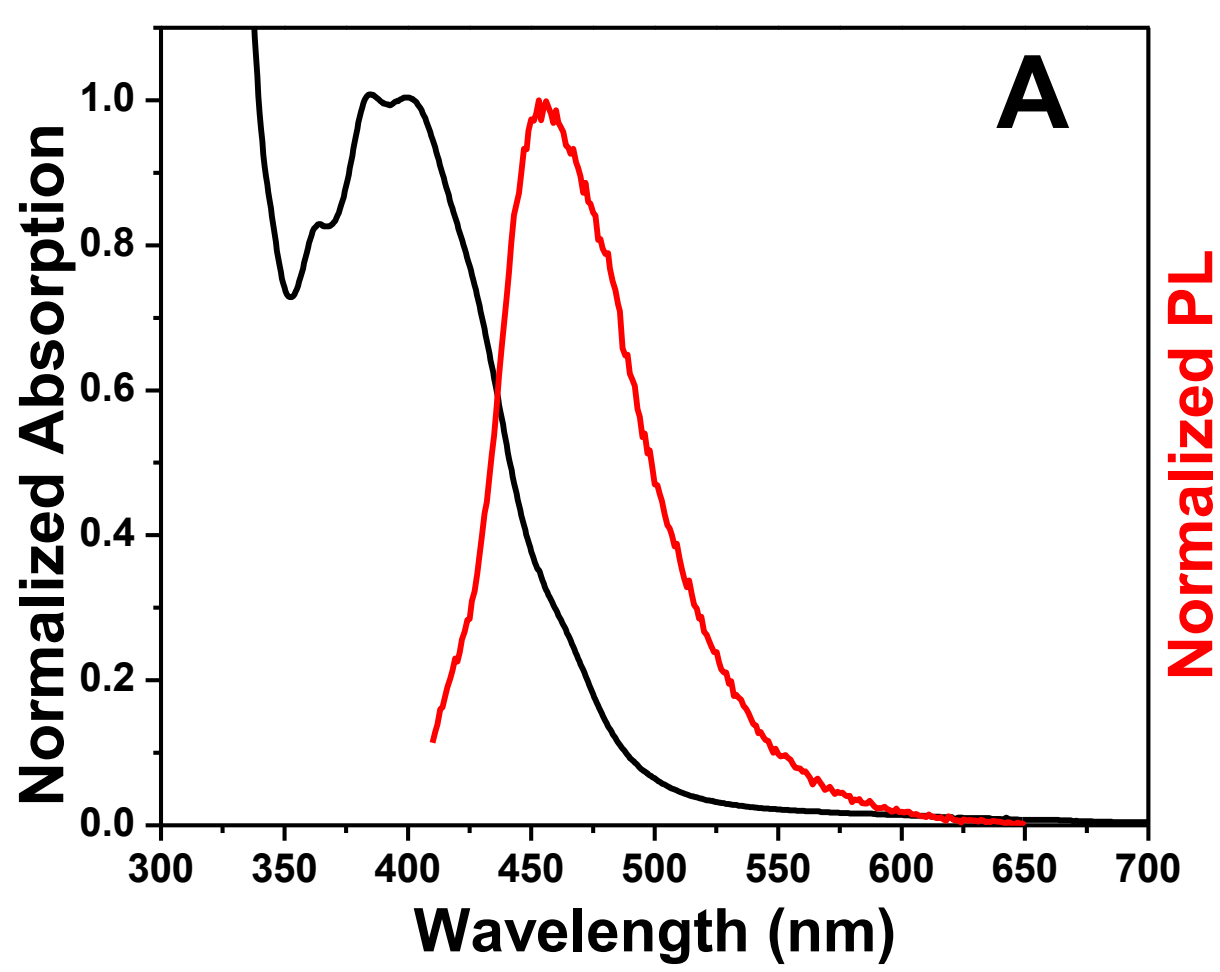

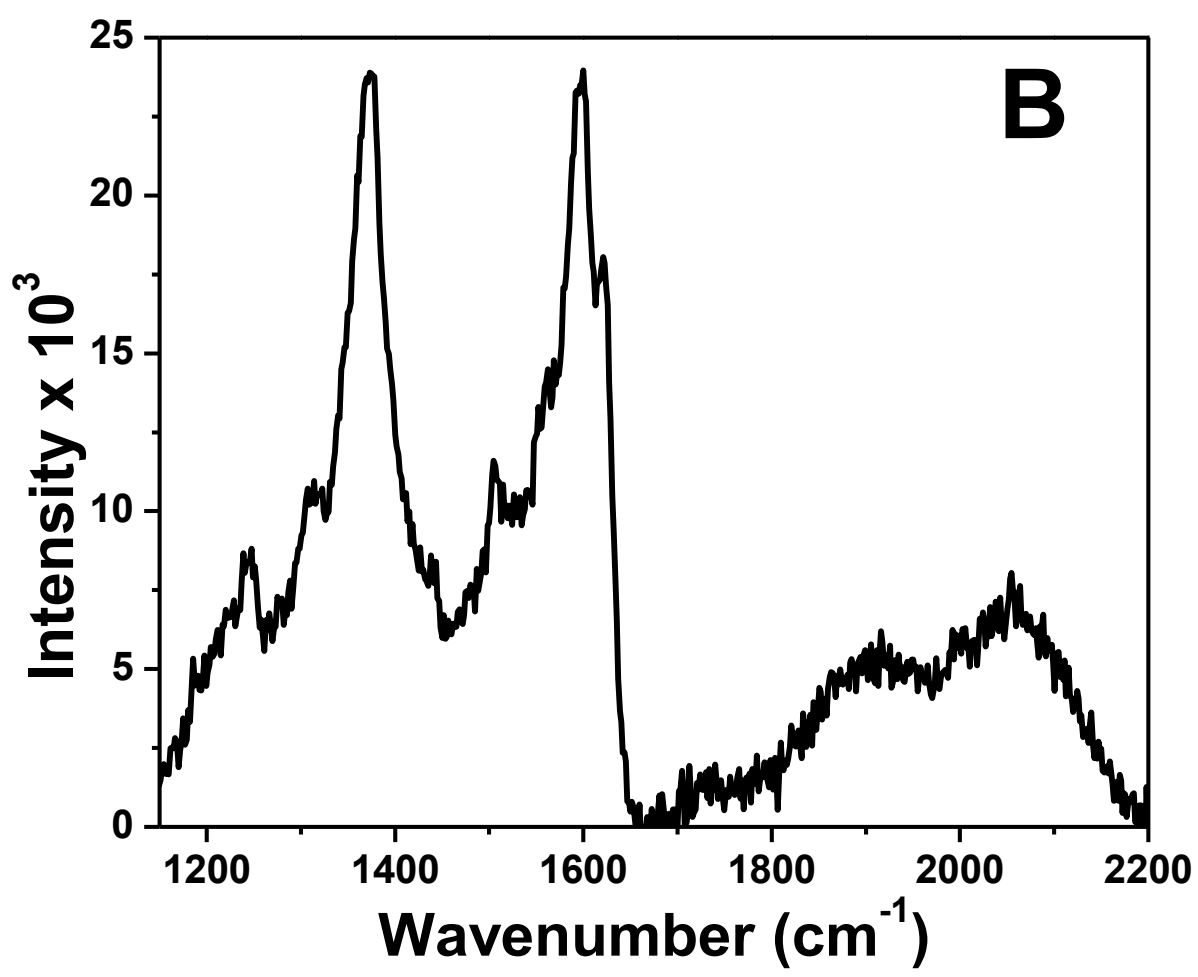

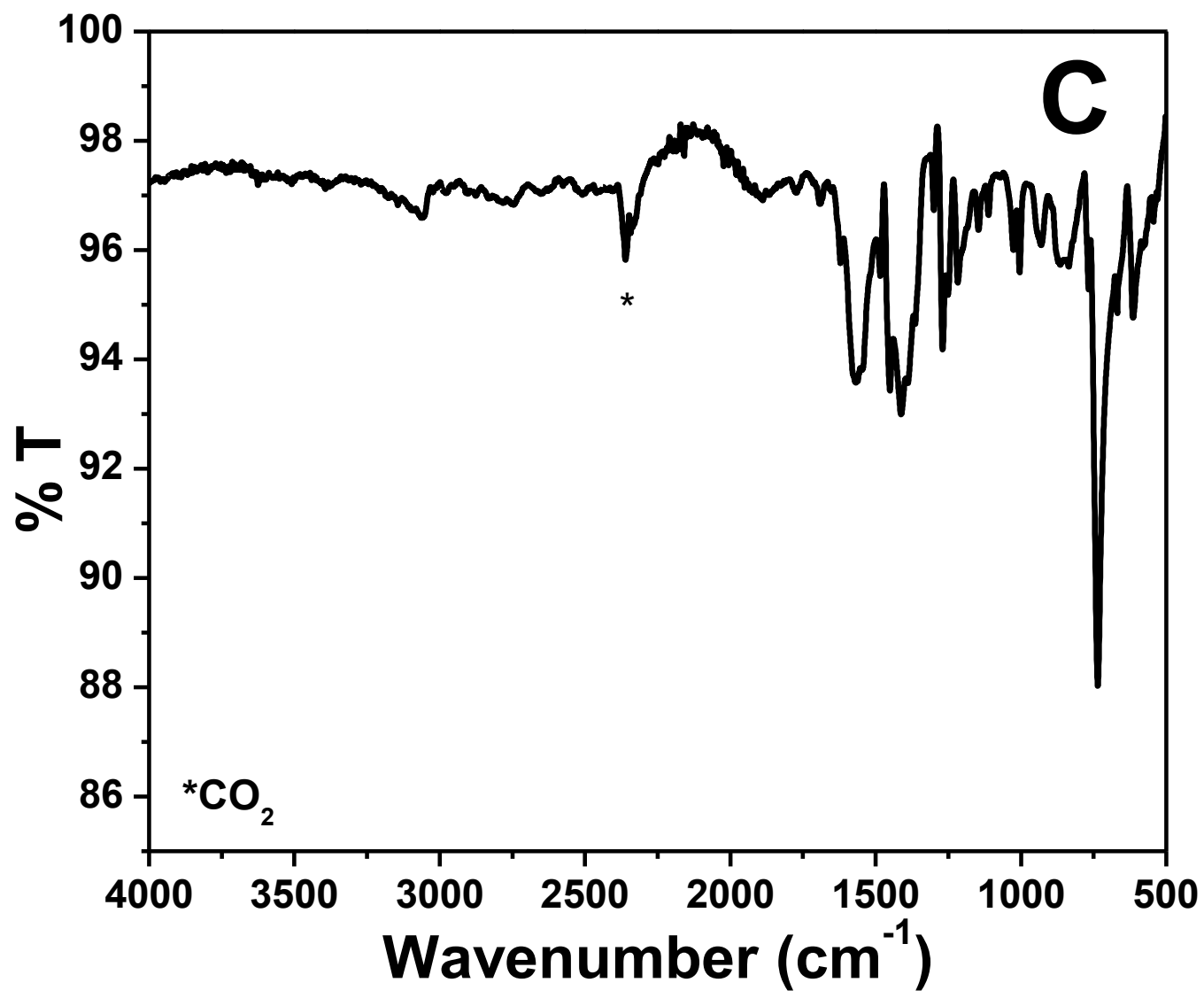

Figure S3. (A) Optical absorption, (B) Raman, and (C) FTIR of CD^-_{Cyan}.

6. X-ray Photoelectron Spectroscopy (XPS) of Synthesized Carbon Dots

The surface elemental compositions of CD^-_{Pyr} and CD^-_{Cyan} were interrogated by X-ray photoelectron spectroscopy (XPS), with results summarized in Figures S4 and S5, respectively. Survey spectra for both samples confirm the presence of carbon, nitrogen, and oxygen as the principal surface elements. Quantitative analysis of CD^-_{Pyr} (Figure S4 A) yields atomic concentrations of 61.75% carbon and 5.14% nitrogen, while CD^-_{Cyan} (Figure S5 A) shows 63.42% carbon and 5.73% nitrogen, confirming the nitrogen-doped, carbon-rich character of both materials.

High-resolution C 1s deconvolution of CD^-_{Pyr} (Figure S4 B) resolves three chemically distinct carbon environments at 284.8 eV (sp^2 C=C), 286.3 eV (C–O), and 288.2 eV (C=O). For CD^-_{Cyan} (Figure S5 B), the C 1s envelope was similarly deconvoluted into peaks at 284.9 eV (sp^2 C=C), 286.0 eV (C–O), and 288.5 eV (C=O), with two additional higher binding energy components at

293.0 and 296.0 eV attributable to carboxylated carbonyl species, consistent with the carboxylic acid-rich surface chemistry expected from the citric acid precursor.

The N 1s spectrum of CD^{-}_{Pyr} (Figure S4 C) was deconvoluted into two components at 400.47 eV (C–N sp^3) and 402.87 eV (N–H), while the N 1s spectrum of CD^{-}_{Cyan} (Figure S5 C) yields components at 400.0 eV (C–N sp^3) and 401.7 eV (N–H), reflecting the nitrogen functionalities introduced by the o-phenylenediamine precursor in the CD^{-}_{Cyan} synthesis.

Deconvolution of the O 1s spectrum of CD^{-}_{Pyr} (Figure S4 D) reveals two contributions at 532.2 eV and 535.4 eV, assigned to C=O/ O–C=O and isolated hydroxyl (–OH) environments, respectively. The O 1s spectrum of CD-Cyan (Figure S5 D) is broader and resolves into three components at 531.1 eV (C=O/O–C=O/isolated –OH), 532.5 eV (C=O), and 535.8 eV (–OH), reflecting the greater abundance and diversity of oxygen-containing functional groups on the CD^{-}_{Cyan} surface arising from the oxygen-rich citric acid precursor. Taken together, the XPS data establishes that CD^{-}_{Pyr} is predominantly amine-functionalized with a graphitic carbon core, whereas CD^{-}_{Cyan} presents a carboxylate- and hydroxyl-rich surface — a chemically consistent outcome given the respective molecular precursors employed in each synthesis.[4]

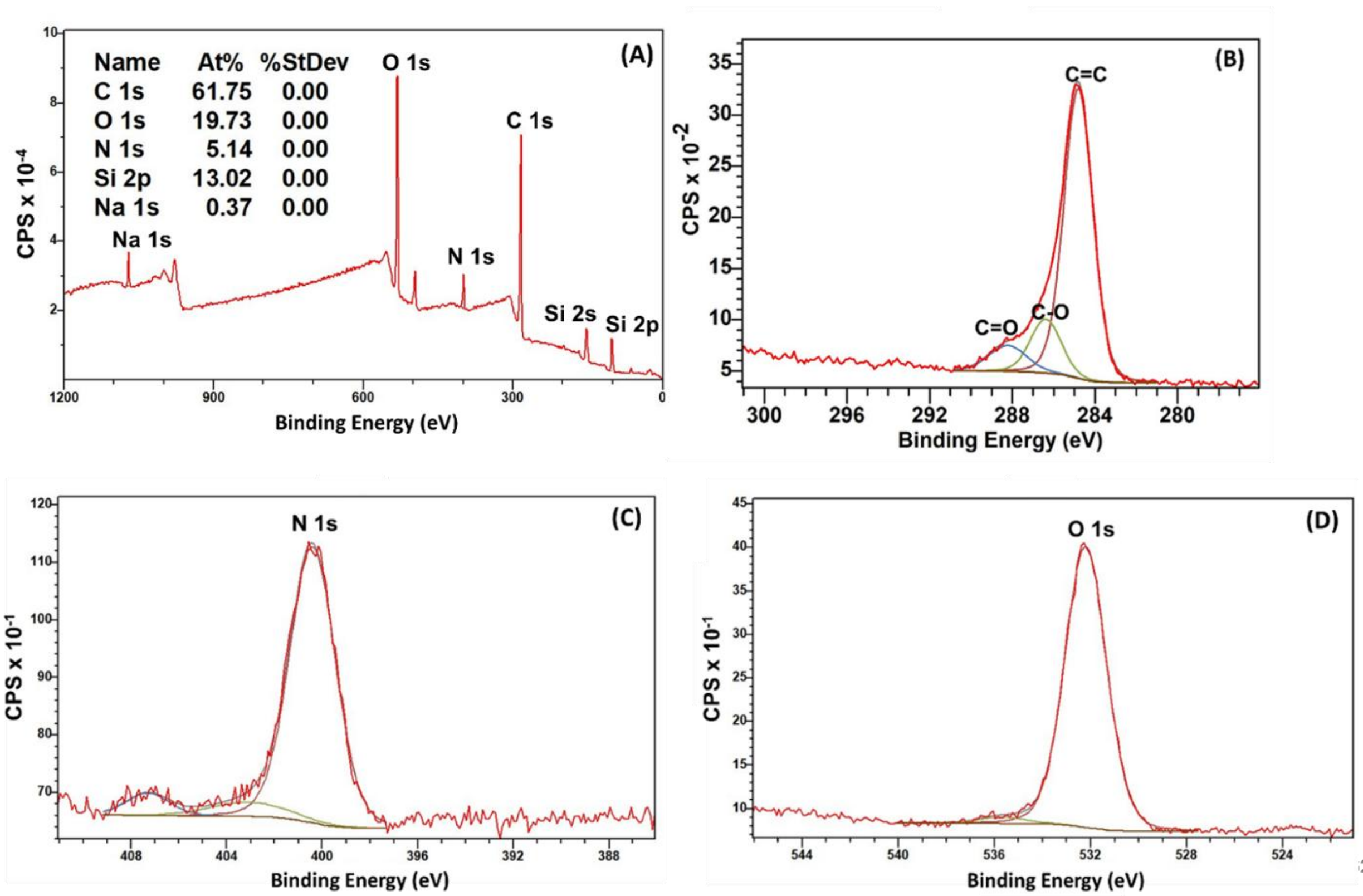

Figure S4. (A) XPS spectra of CD^{-}_{Pyr} show carbon, nitrogen, and oxygen peaks, (B) Deconvoluted C 1s, (C) N 1s, and (D) O 1s spectrum.

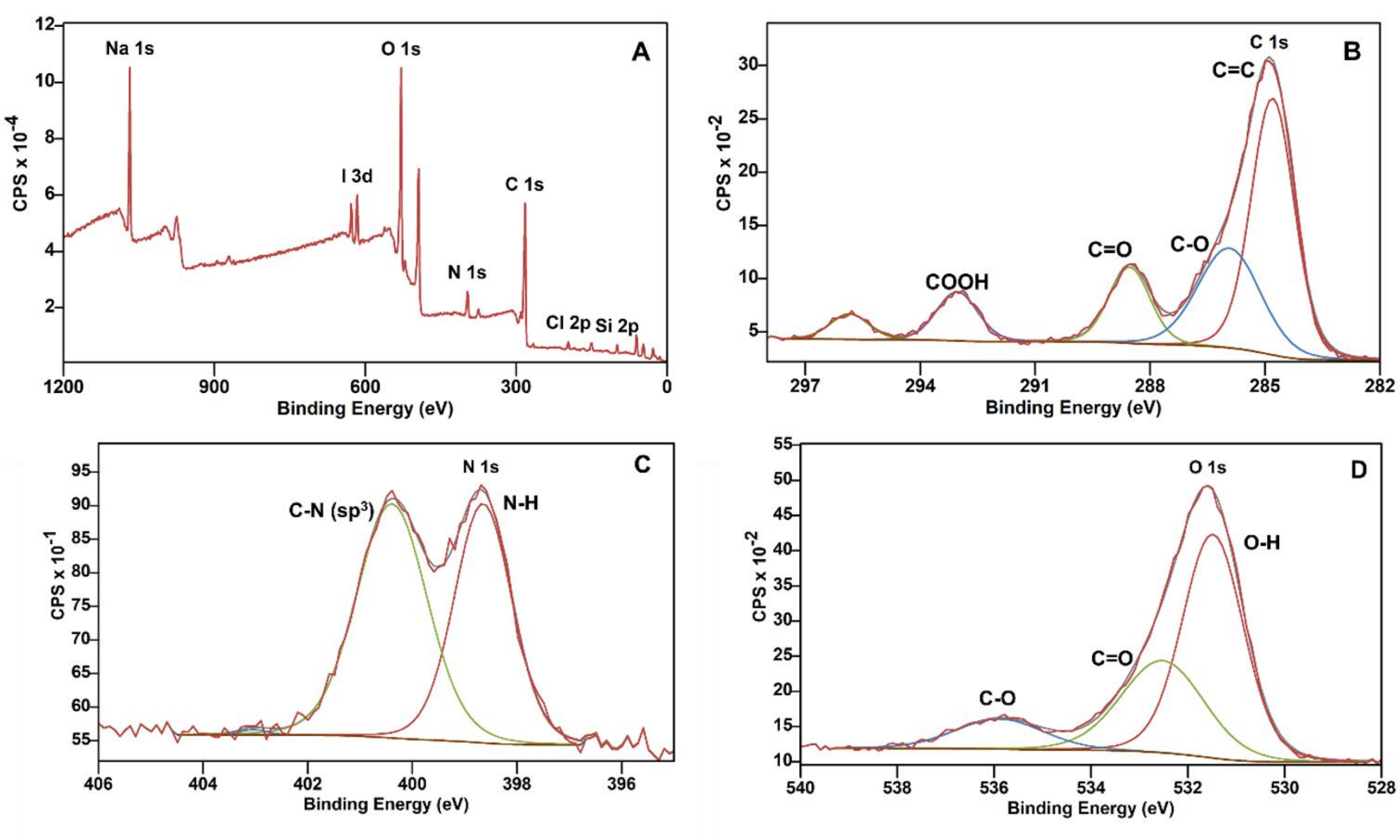

Figure S5. (A) XPS spectra of CD^-_{Cyan} show carbon, nitrogen, and oxygen peaks, (B) Deconvoluted C 1s, (C) N 1s, and (D) O 1s spectra.

7. Time-Resolved PL Kinetics Decay Traces

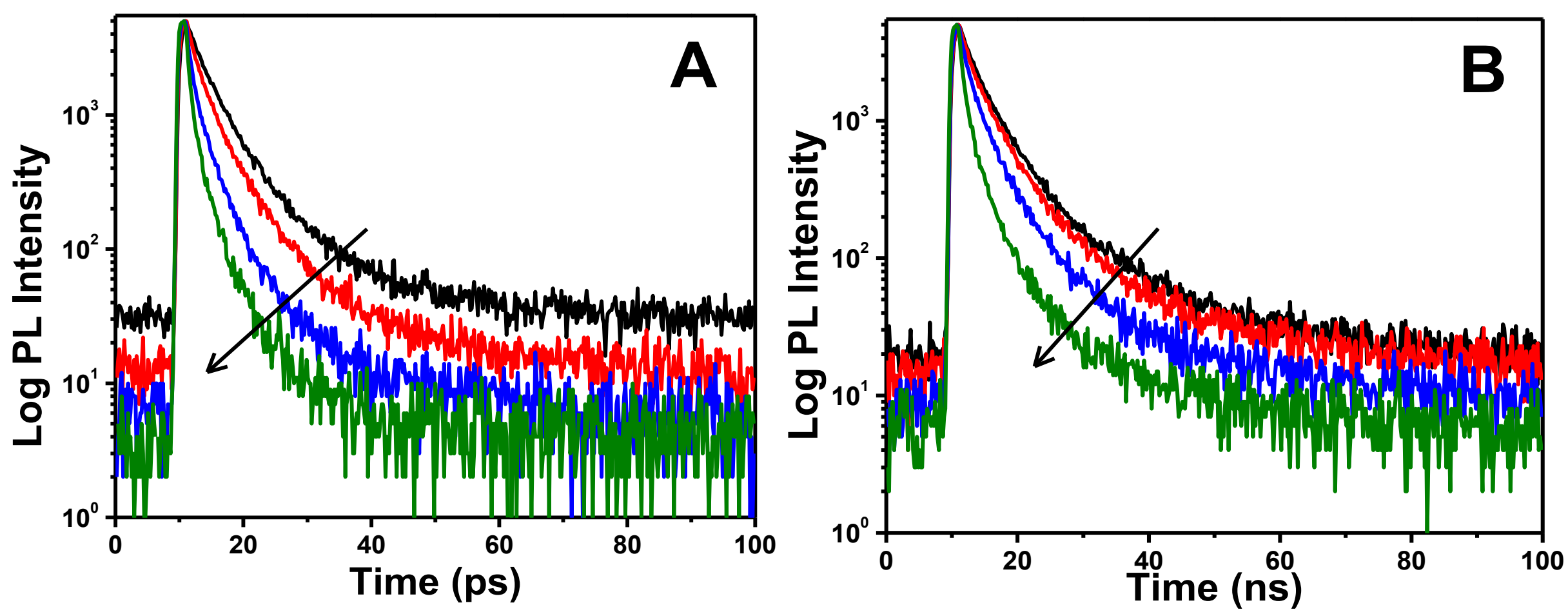

Figure S6. PL kinetic decay traces of $CsPbBr_3$ with different concentrations of (A) CD^-_{Pyr}, and (B) CD^-_{Cyan}.

Table S1. Time-resolved PL decay parameters of pure $CsPbBr_3$ nanocrystals and (A) $CsPbBr_3$/ CD^-_{pyr} and (B) $CsPbBr_3$/ CD^-_{Cyan} (λ_{ex}=370 nm and λ_{em}= 506 nm).

(A) Sample (CD^-_{pyr})	τ_1 (ns)	τ_2 (ns)	τ_3 (ns)	τ_{avg} (ns)	χ^2
**0 mg/ L**	1.6 ± 0.17 (12.93%)	4.7 ±0.2 (67.06%)	17.0 ± 0.1 (20.01%)	11.0	1.0
**0.69 mg/ L**	1.4 ± 0.08 (25.53%)	4.4 ± 0.17 (64.12%)	16.0 ± 0.1 (10.35%)	8.0	1.0
**2.0 mg/ L**	0.78 ± 0.06 (33.56%)	3.1 ± 0.90 (56.94%)	13.0 ± 0.4 (9.50%)	7.0	1.2
**3.3 mg/ L**	0.53± 0.05 (44.05%)	2.1± 0.05 (43.84%)	7.0 ± 0.5 (12.12%)	4.0	1.3

(B) Sample (CD^{-}_{Cyan})	τ_1 (ns)	τ_2 (ns)	τ_3 (ns)	τ_{avg} (ns)	χ^2
**0 mg/ L**	1.6 ± 0.17 (12.93%)	16.7 ± 0.5 (20.01%)	4.7 ±0.2 (67.06%)	11.0	1.0
**0.69 mg/ L**	1.3 ±0.12 (13.55%)	14.2 ±1 (19.73%)	4.3 ±0.17 (66.72%)	9.0	1.1
**2.0 mg/ L**	1.3 ± 0.06 (29%)	4.2 ±0.2 (59.19%)	16 ±1.7 (11.81%)	8.5	1.0
**3.3 mg/ L**	0.68 ±0.02 (39.75%)	2.8 ±0.1 (47.65%)	13.0 ±0.99 (12.60%)	8.0	1.0

8. FTIR Measurements

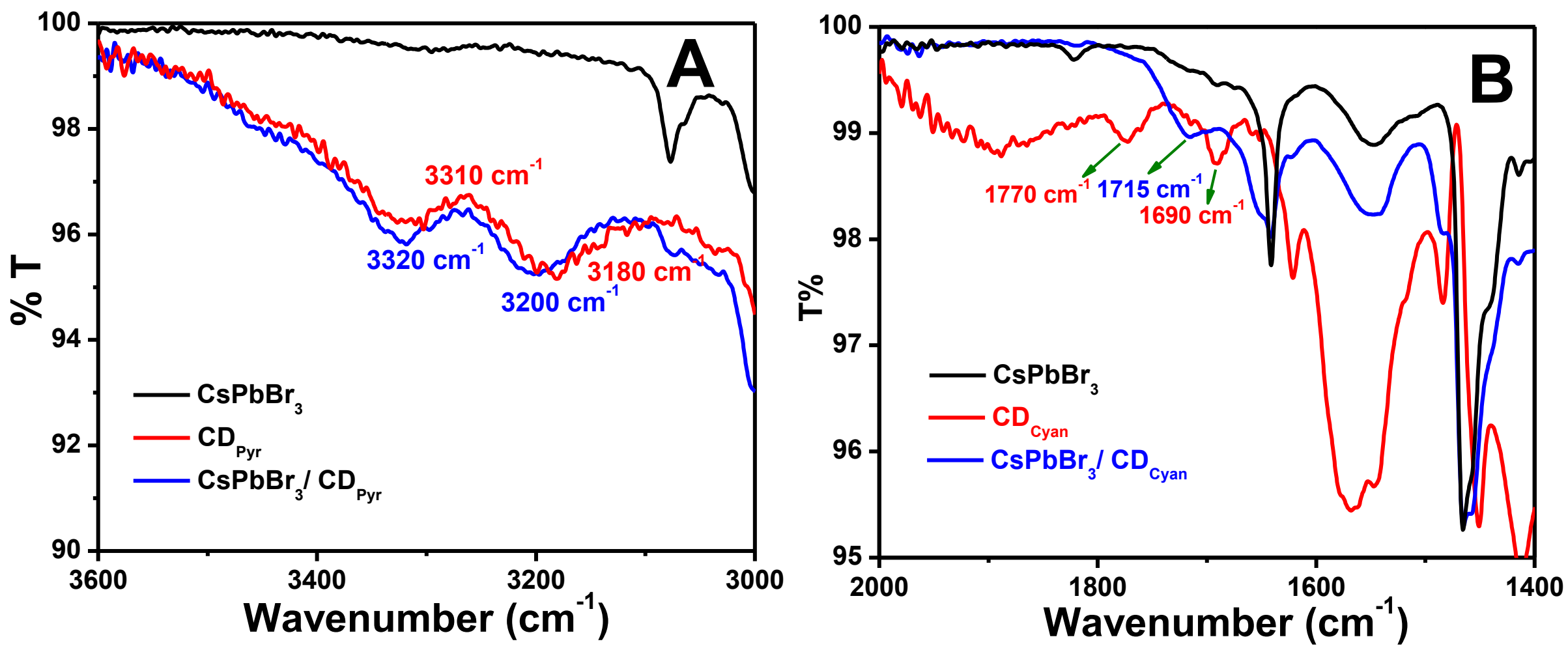

Figure S7. Comparison of FTIR spectra of $CsPbBr_3$, CD, and $CsPbBr_3$/CD hybrids for (A) CD^{-}_{Pyr}, and (B) CD^{-}_{Cyan}.

9. Transient Absorption Measurement

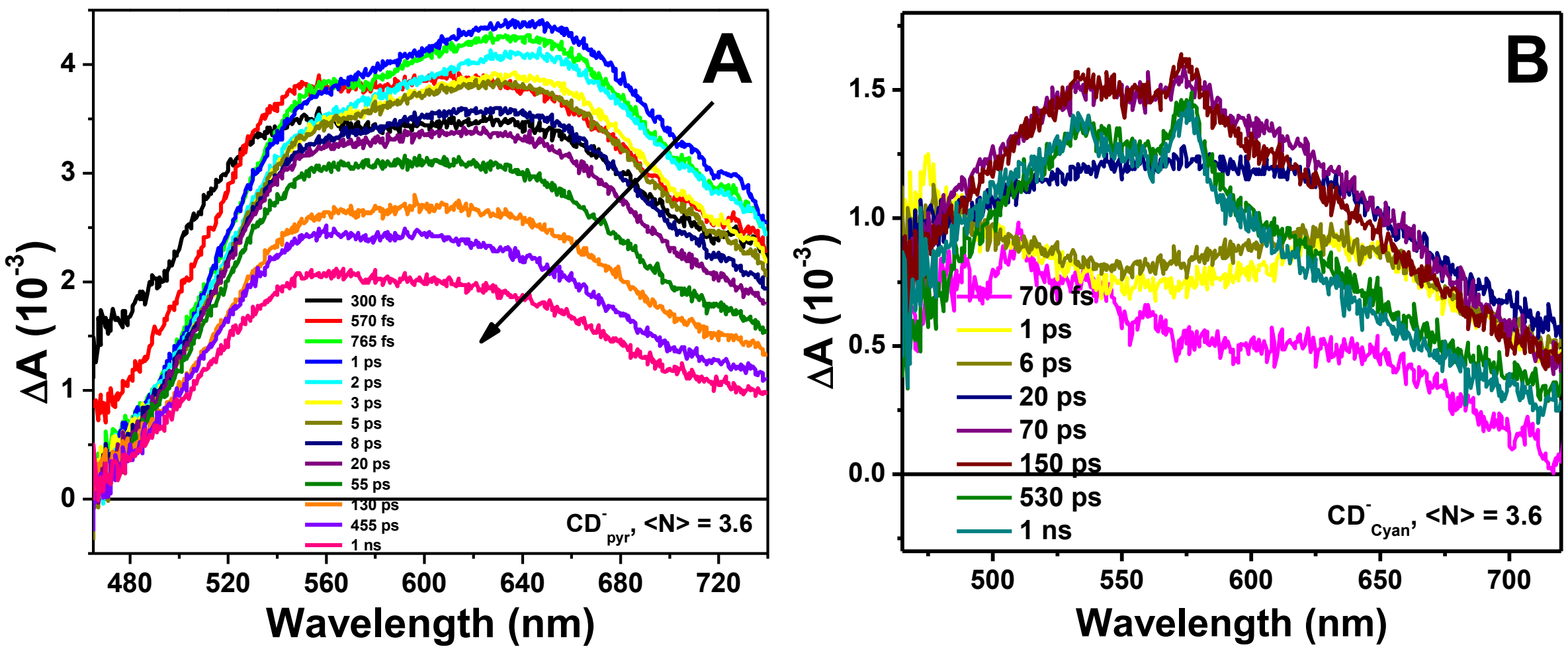

Figure S8. Excited-state absorption spectra at different time delays of (A) CD^{-}_{Pyr}, (B) CD^{-}_{Cyan} in toluene at 370 laser excitation, <N> = 3.6.

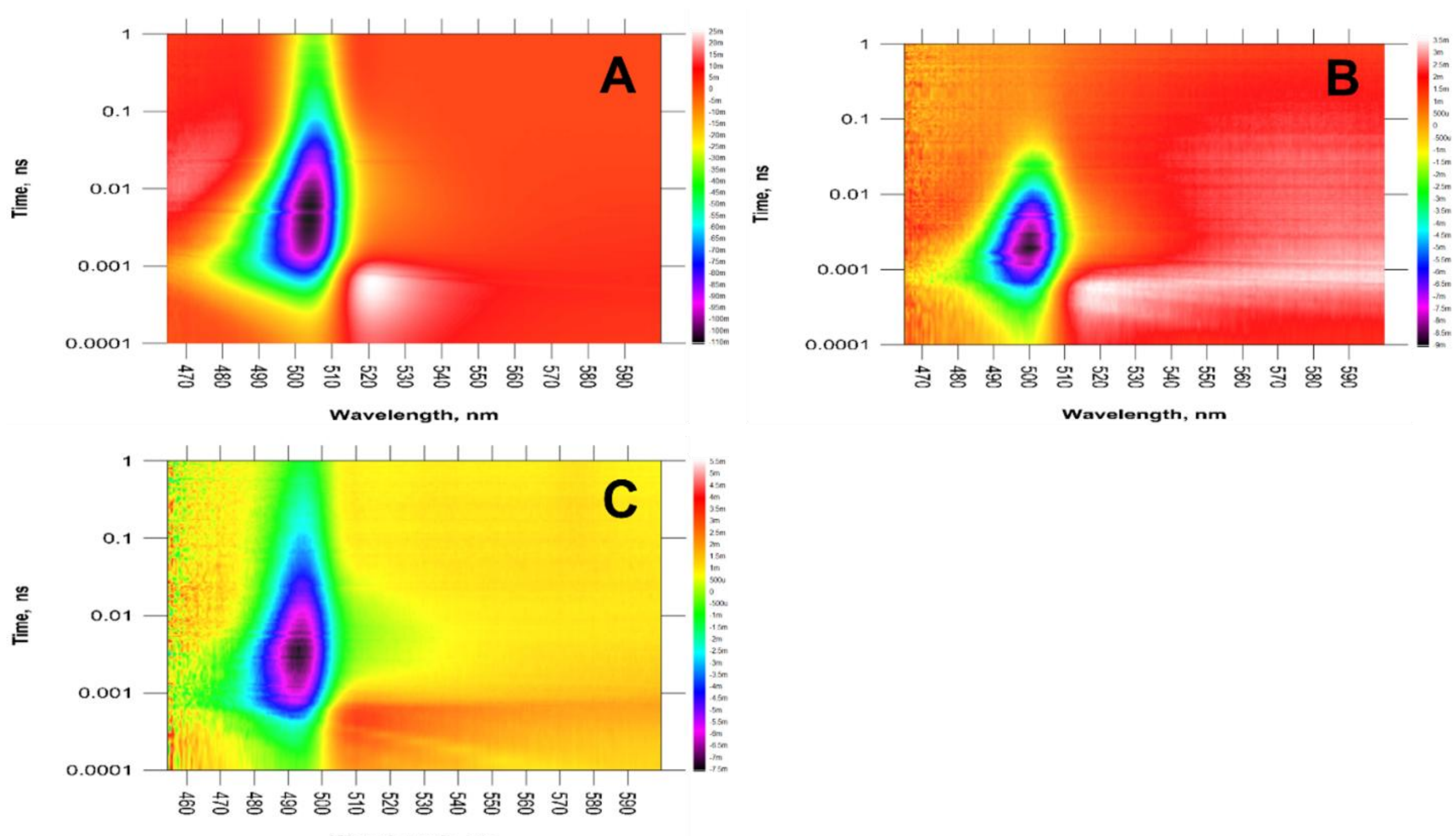

Figure S9. Transient absorption color mapping of (A) $CsPbBr_3$, (B) $CsPbBr_3/CD^{-}_{Pyr}$, and (C) $CsPbBr_3/CD^{-}_{Cyan}$ at high pump fluence (<N> = 2.2).

10. Bleach Recovery Kinetics

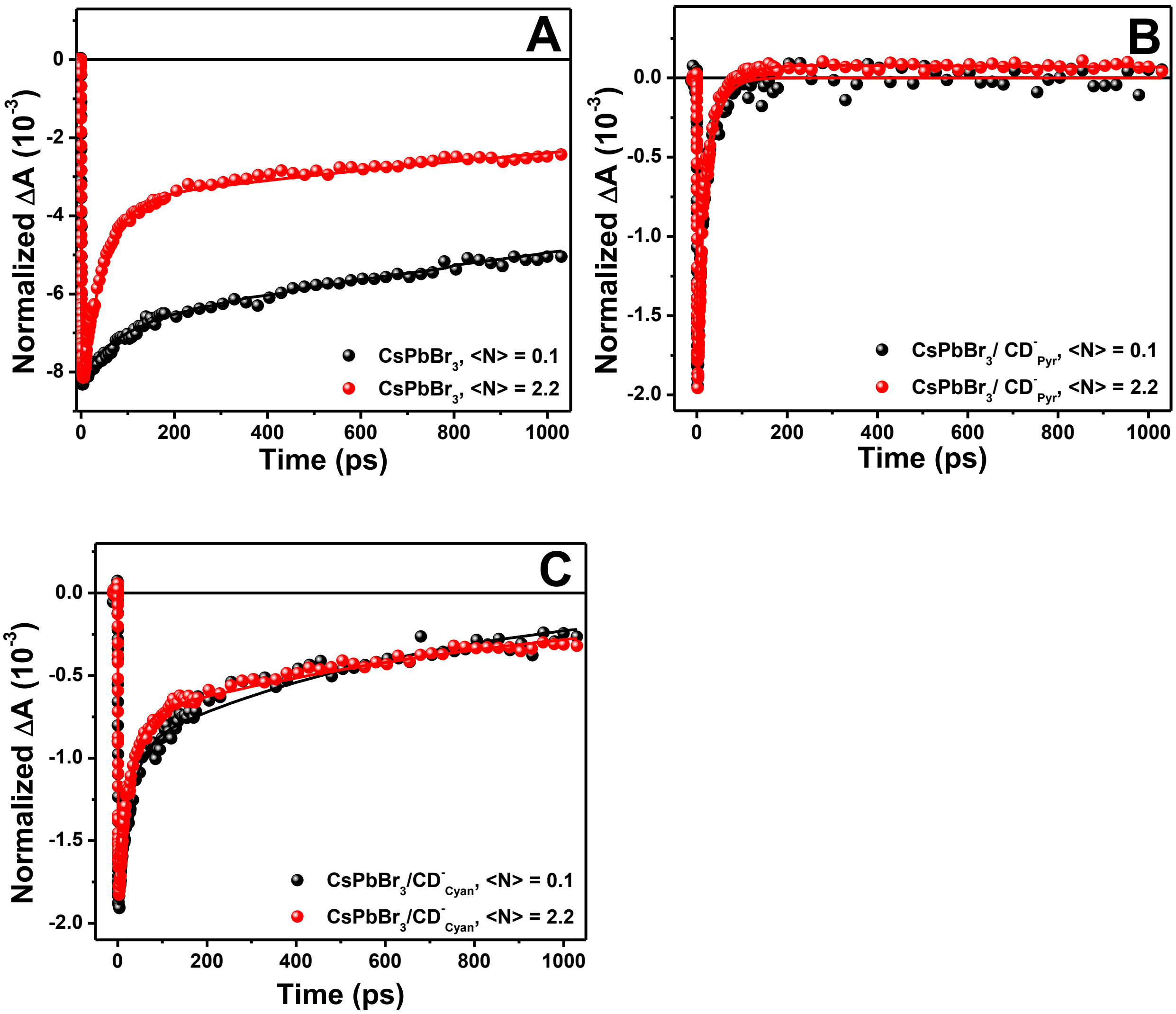

Figure S10. Comparative Analysis of Bleach Recovery Kinetics in $CsPbBr_3$, $CsPbBr_3$/ CD^-_{Pyr}, and $CsPbBr_3$/ CD^-_{Cyan} under different pump fluences.

11. Bleach Formation Kinetics

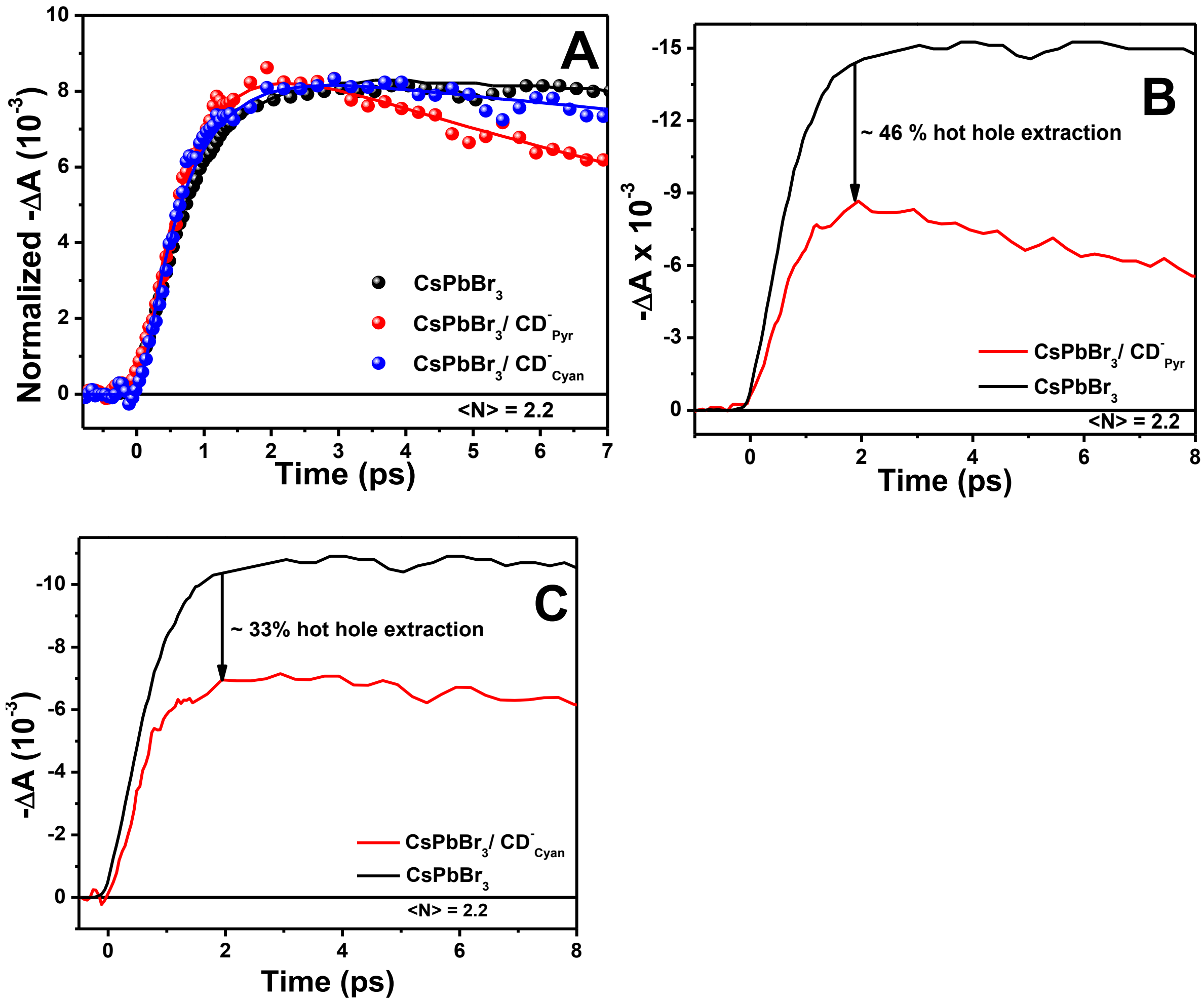

Figure S11. (A) Comparison of the bleach formation kinetics, and (B, C) Absolute bleach amplitude comparison at early time scales between $CsPbBr_3$, $CsPbBr_3$/ CD^-_{Pyr} and $CsPbBr_3$/ CD^-_{Cyan}. λ_{ex}=370 nm and <N> = 2.2.

Table S2. Bleach formation time for $CsPbBr_3$ NCs, $CsPbBr_3$/ CD^-_{Pyr} and $CsPbBr_3$/ CD^-_{Cyan} NCs at 370 nm laser excitation and different pump powers.

Sample	τ_{Hot} (ps)	τ_{CT} (ps)	τ_{Recomb} (ps)	τ_{Decay} (ns)
$CsPbBr_3$, <N> = 0.1	0.55 ± 0.1 (growth)	-	-	> 1 (100%)
$CsPbBr_3$/ CD^-_{Pyr}, <N> = 0.1	0.32 ± 0.1 (growth)	8 ± 1 (-61.2%)	42 ± 5 (-38.8%)	-

$CsPbBr_3$/ CD^-_{Cyan}, <N> =0.1	0.38 ± 0.1 (growth)	27 ± 3(-50%)	679 ± 30 (-50%)	-
$CsPbBr_3$, <N> = 2.2	0.80 ± 0.1 (growth)	45 ± 5 (-58.0%)	-	>1 (-42%)
$CsPbBr_3$/ CD^-_{Pyr}, <N> = 2.2	0.60 ± 0.2 (growth)	10 ± 3 (-61.6%)	42 ± 10 (-34.4%)	>(4.0%)
$CsPbBr_3$/ CD^-_{Cyan}<N> = 2.2	0.62 ± 0.1 (growth)	27 ± 3 (-58.1%)	679 ± 30 (-32.9%)	>1 (-9%)

12. Species-Associated Spectra

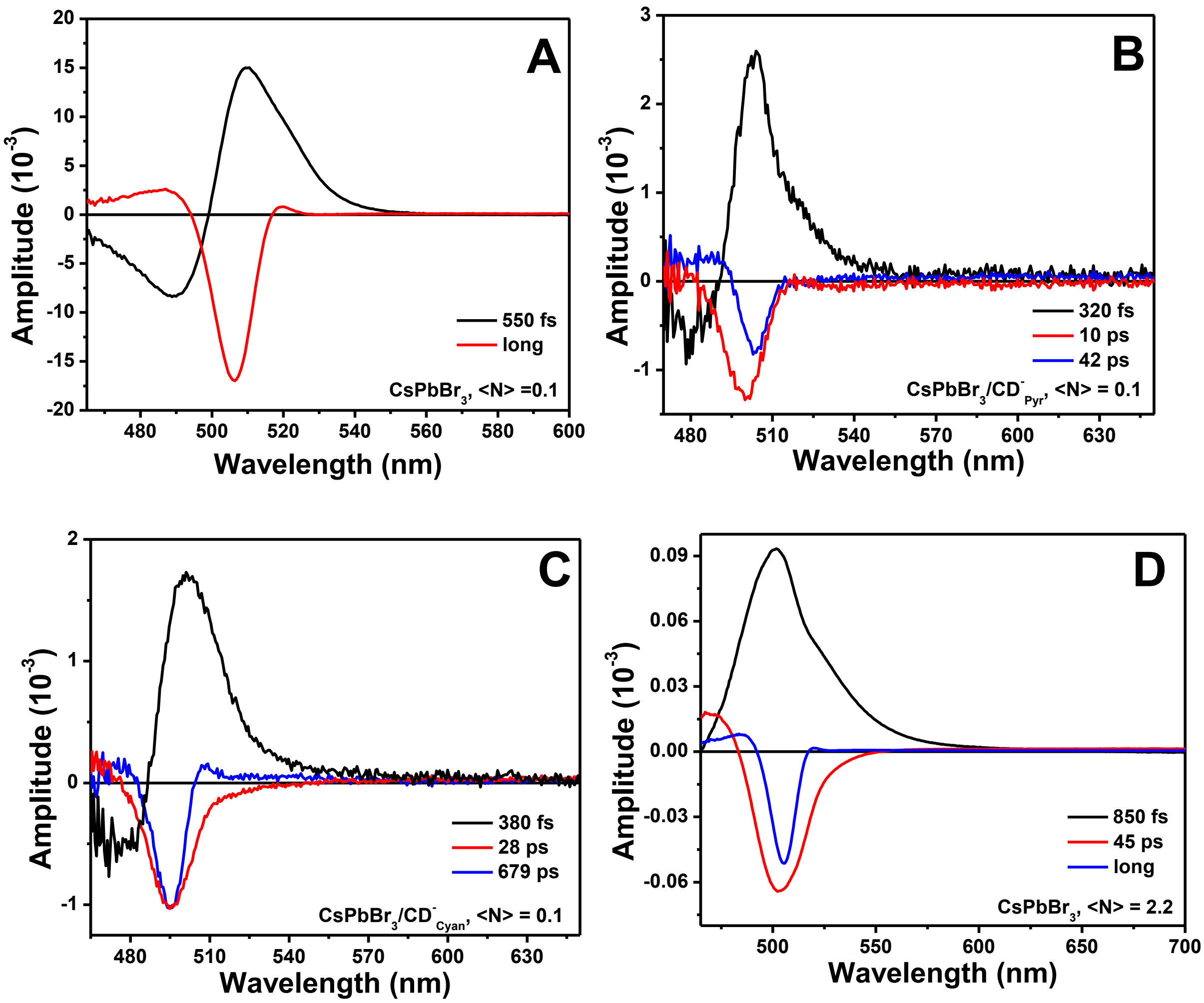

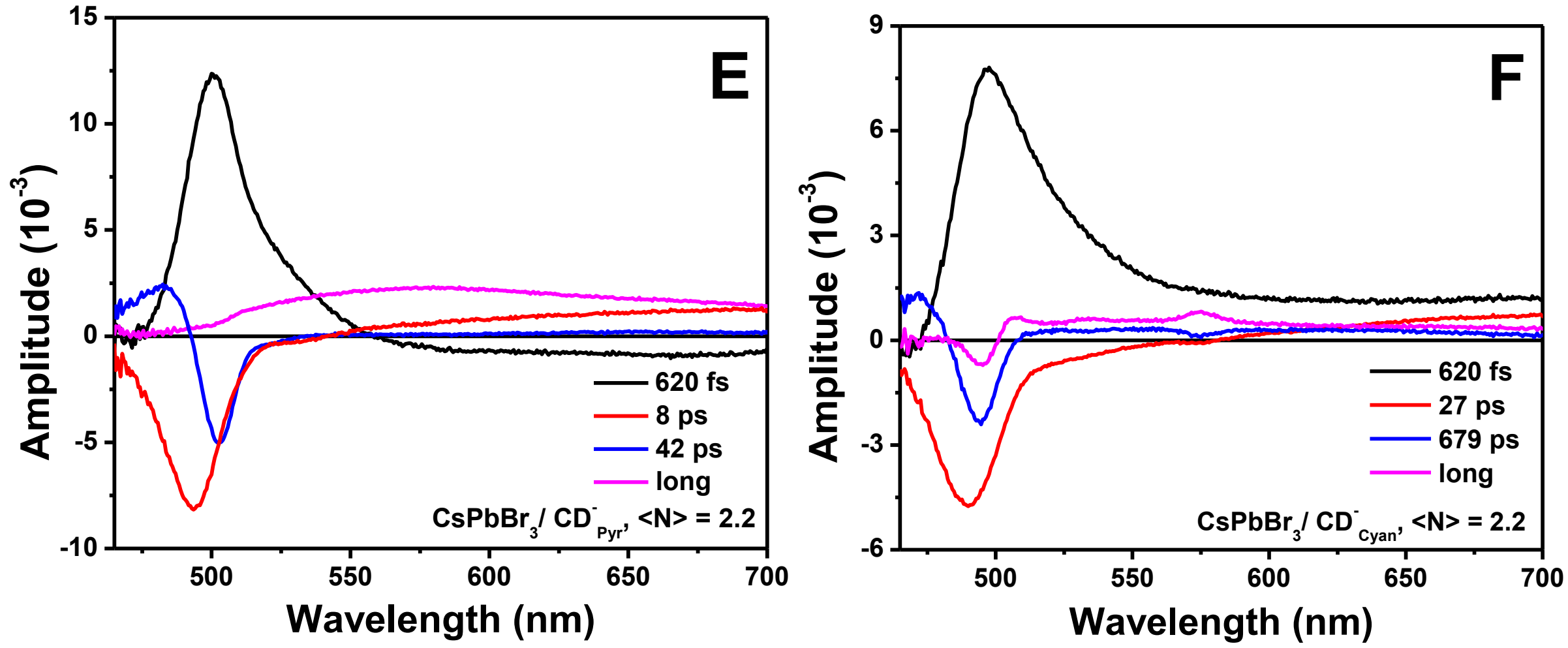

Figure S12. Species-associated spectra obtained from global fit analysis at low and high pump power for (A, D) $CsPbBr_3$, (B, E) $CsPbBr_3/CD^-_{Pyr}$, and (C, F) $CsPbBr_3/CD^-_{Cyan}$.

13. Cyclic Voltammetry (CV)

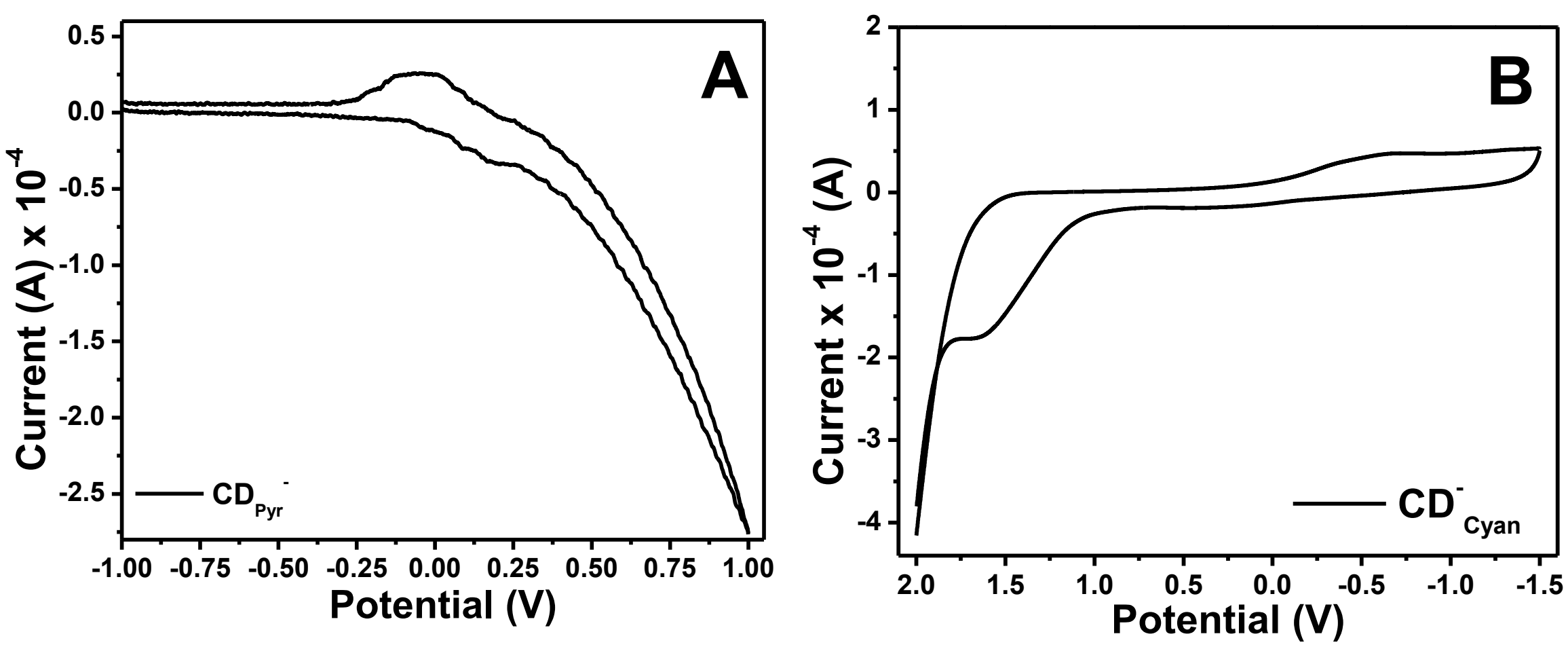

Figure S13. Cyclic voltammetry of (A) CD^-_{Pyr}, and (B) CD^-_{Cyan}

Calculations

1. Band gap of CD^-_{pyr} (E_g) = 2.80 eV

The reduction potential of CD^-_{pyr} = (-0.050+ 0.024)/ 2 = -0.013 V vs Ag/AgCl/Cl-

= (0.197 + (-0.014)) = (+) 0.183 V vs NHE

Oxidation potential of CD^-_{pyr} = (-) 0.183 V = (-4.74-0.183) vs vacuum = -4.9 eV

E_{HOMO} = -4.9 eV

E_{LUMO} = 2.80 + (-4.9) = -2.1 eV

2. Band gap of CD^{-}_{cyan} (E_g) = 2.99 eV

Since the CV of CD^{-}_{cyan} is not reversible, the reduction potential of CD^{-}_{cyan} = 1.6 V = (+ 0.197) + (1.6) = (+) 1.897 V vs NHE

The value of 1.897 V (versus NHE) in eV is given by: – 4.74 eV (≅ 0 V versus NHE) – 1.897 = -6.6 eV.

E_{HOMO} = -6.6 eV

E_{LUMO} = 2.99 + (- 6.6) = -3.6 eV

14. Spectral Overlap of Donor Emission and Acceptor Absorption

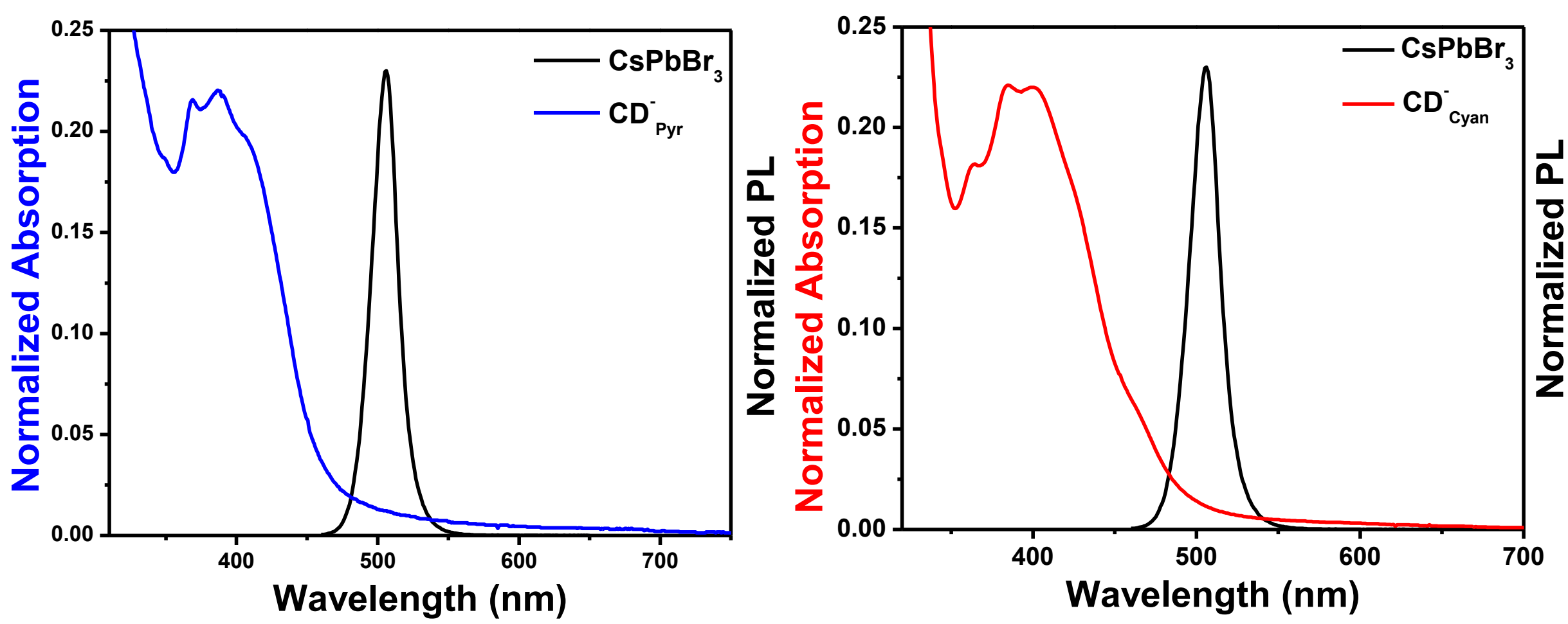

Figure S14. Spectral overlap between $CsPbBr_3$ emission and CD absorption.

15. Calculation of average number of absorbed photons per nanocrystal

The average number of absorbed photons per nanocrystal was determined to be around 0.1 to 2.2, and the calculation follows:

$$< N > = \sigma . j \ldots \ldots \ldots \ldots$$

Where, j is the pump fluence, i.e. number of photon/cm^2 and σ= absorption cross section Pump energy used =1 μJ/cm^2.

Pump fluence (j) = Pump energy (J)/ (π (spot size)^2) ---- (3)

Pump frequency = 2.5 kHz

Spot size = 500 micron

At 300 μW pump power, Pump Energy (J) = Pump power (W) x time (s) = (300 x 10^{-6} x (10^{-3}/2.5)) W.s = 1.2 x 10^{-7} J

Using equation (3), Pump fluence = 1.2 x 10^{-7} J/ (π x (0.05 cm)^2) = 15 μJ/cm^2

Similarly, at 10 μW pump power, Pump Energy = (10 x 10^{-6} x (10^{-3}/2.5)) W.s = 4 x 10^{-9} J

Pump fluence = 4 x 10^{-9} J/ (π x (0.05 cm)^2) = 0.51 μJ/cm^2

Excitation frequency (u) = $\frac{Velocity\ of\ light\ (m/s)}{Excitation\ wavelength\ (nm)} = \frac{3\ x\ 10^{8}\ m/s}{370\ x\ 10^{-9} m} = 8.1\ x\ 10^{14}\ s^{-1}$

<N> = $\frac{15\ x\ 10^{-6}}{6.626\ x\ 10^{-34}\ x\ 8.1\ x\ 10^{14}}\ x\ 7.8\ x\ 10^{-14} = 2.17\ \approx\ 2.2\ (@300\ \mu W)$

<N> = $\frac{0.51\ x\ 10^{-6}}{6.626\ x\ 10^{-34}\ x\ 8.1\ x\ 10^{14}}\ x\ 7.8\ x\ 10^{-14} = 0.09\ (@10\ \mu W)$ ≈ 0.1

Considering the absorption cross-section of $CsPbBr_3$ from previous literature, [5] σ = 7.8 x 10^{-14} cm^2.

References

1. L. Protesescu, S. Yakunin, M. I. Bodnarchuk, F. Krieg, R. Caputo, C. H. Hendon, R. X. Yang, A. Walsh and M. V. Kovalenko, *Nano Lett.*, 2015, **15**, 3692–3696.

2. A. Madonia, M. Martin-Sabi, A. Sciortino, S. Agnello, M. Cannas, S. Ammar, F. Messina and D. Schaming, *J. Phys. Chem. Lett.*, 2020, **11**, 52.
3. B. Wang, J. Yu, L. Sui, S. Zhu, Z. Tang, B. Yang and S. Lu, *Adv. Sci.*, 2021, **8**, 2001453.
4. A. Dager, T. Uchida, T. Maekawa and M. Tachibana, *Sci. Rep.*, 2019, **9**, 14004.
5. Chen, J.; Messing, M. E.; Zheng, K.; Pullerits, T. Cation-Dependent Hot Carrier Cooling in Halide Perovskite Nanocrystals. *J. Am. Chem. Soc.* **2019**, *141* (8), 3532–3540.
